# Multidimensional targeting of ischemia-reperfusion injury by genistein: from molecular crosstalk to clinical translation

**DOI:** 10.3389/fphar.2026.1864013

**Published:** 2026-06-17

**Authors:** Ruihua Wang, Chunye Ya, Wei He, Yunjian Pan, Cunguo Shi, Yujing Lin, Xuekun Xing

**Affiliations:** 1 School of Public Health, Guilin Medical University, Guilin, China; 2 Intensive Care Unit, Liuzhou Workers’ Hospital, LiuZhou, China; 3 Guangxi Key Laboratory of Environmental Exposomics and Entire Lifecycle Heath, Guilin, China; 4 Guangxi Key Laboratory of Entire Lifecycle Health and Care, Guilin, China

**Keywords:** apoptosis, genistein, inflammatory response, ischemia-reperfusion injury, oxidative stress, pharmacokinetics, translational pharmacology

## Abstract

Ischemia-reperfusion injury (IRI) is a convergent pathology driven by oxidative stress, sterile inflammation, mitochondrial dysfunction, and regulated cell death (apoptosis, necroptosis, pyroptosis, ferroptosis), yet validated pharmacotherapies remain scarce. Genistein, a soy-derived isoflavone phytoestrogen, has demonstrated multi-organ protection in preclinical IRI models through coordinated regulation of the Nrf2/HO-1 antioxidant axis, SIRT1/p53 deacetylation-dependent anti-apoptotic signaling, and NF-kappaB/JAK2-STAT3/alpha7nAChR/NLRP3 inflammasome cascades, but systematic mechanistic integration is lacking. A narrative review with systematic literature identification was conducted using PubMed/MEDLINE, Web of Science, and Scopus (2010–2024). Studies on genistein or its structurally defined derivatives in established IRI models with mechanistic endpoints were included; soy extracts, biochanin A, and non-IRI studies were excluded. Genistein engages multiple cytoprotective pathways with organ-dependent evidence strength. Causal validation (Level A: genetic deletion, siRNA, or pharmacological inhibitor with rescue) has been achieved for Nrf2/HO-1 in cerebral IRI and for SIRT1/p53, ADORA2A-cAMP-PK, and PI3K/Akt in renal IRI, whereas hepatic and intestinal evidence remains correlative (Level C). SIRT1-mediated deacetylation concurrently suppresses both p53-dependent apoptosis (Bax/PUMA) and NF-kappaB p65 subunit transcriptional activity at Lys310, integrating anti-apoptotic and anti-inflammatory effects. Genistein inhibits NLRP3 inflammasome activation at the priming level (NF-kappaB-dependent NLRP3/pro-IL-1beta transcription) and assembly level (ROS/ASC/caspase-1), linking oxidative stress sensing to gasdermin D-mediated pyroptosis. Emerging evidence (2023–2025) suggests genistein may attenuate ferroptosis via iron chelation and Nrf2-driven GPX4 preservation. Translational gaps include concentration disconnect between *in vitro* effective doses (10–100 μM) and *in vivo* free aglycone levels (<0.1 μM), unaddressed PAINS assay-interference liability, predominance of pretreatment-only rodent models, undefined drug interaction profiles, and absence of comorbidity-rich and large-animal studies. Genistein-3′-sodium sulfonate demonstrates improved aqueous solubility and post-treatment efficacy in cerebral IRI, but human pharmacokinetic data in IRI contexts are absent. Genistein’s multi-target, cross-pathway pharmacology aligns with IRI pathophysiology, yet the evidence base is insufficiently rigorous for clinical deployment. This review identifies SIRT1-mediated dual p53/NF-kappaB suppression as a mechanistic nexus, highlights pyroptosis and ferroptosis as underexplored therapeutic dimensions, and proposes a translational roadmap prioritizing causal pathway validation with orthogonal PAINS-controlled assays, pharmacokinetic characterization, sex- and comorbidity-stratified preclinical models, and early-phase clinical investigation in elective surgical settings where prophylactic administration is feasible.

## 1 Introduction

Ischemia-reperfusion injury (IRI) occurs when a tissue or organ experiences a period of ischemia, which significantly reduces the oxygen supply and deprives the tissue of energy substrates. When blood flow is suddenly restored, there is an instantaneous increase in oxygen supply to the affected area. This drastic change can trigger severe tissue or organ damage and may exacerbate pre-existing injuries (Barrou et al., 2016; Zhang M. et al., 2024). In the domain of clinical medicine, IRI manifests itself during and after surgical procedures, exhibiting a close correlation with the patient’s recovery process. IRI has been identified as a pivotal factor in various pathologies, including stroke and myocardial infarction, as well as in complications and mortality associated with organ transplantation. This underscores its great clinical significance.

It has been demonstrated that IRI is capable of inducing a variety of detrimental physiological processes, including oxidative stress, inflammation, and apoptosis (Zhang M. et al., 2024). The pathophysiology of IRI converges on several interdependent processes: burst production of reactive oxygen species (ROS) upon reoxygenation, mitochondrial permeability transition pore (mPTP) opening, calcium overload, sterile inflammation driven by damage-associated molecular patterns (DAMPs), endothelial barrier disruption and glycocalyx shedding, microvascular obstruction (“no-reflow”), complement activation, and activation of regulated cell death pathways including apoptosis, necroptosis (RIPK1/RIPK3/MLKL), pyroptosis (caspase-1/GSDMD), and ferroptosis (GPX4/lipid peroxidation) (Zhang M. et al., 2024). Furthermore, IRI has been demonstrated to produce deleterious mediators, including cytokines, fibrogenic factors, nitrogen oxides, and peroxynitrite.

In recent years, significant progress has been made in studying the biological and molecular mechanisms of IRI. However, therapeutic options remain limited, with a paucity of clinically validated protocols or pharmacotherapeutic agents that are effective in mitigating the harmful consequences of IRI. Ischemic preconditioning and postconditioning have shown promise in experimental settings but have proven difficult to translate into consistent clinical benefit. Therapeutic hypothermia, antioxidants (e.g., N-acetylcysteine, vitamin E, MitoQ), complement inhibitors (e.g., eculizumab), anti-inflammatory biologics (e.g., anti-IL-1beta, anti-TNF), and mitochondrial-targeted agents (e.g., cyclosporine A for mPTP inhibition) have largely yielded disappointing or inconclusive results in large clinical trials, underscoring the translational gap between mechanistic understanding and effective therapy (Zhang M. et al., 2024).

Genistein (4′,5,7-trihydroxyisoflavone; molecular formula C15H10O5, molecular weight 270.24 g/mol) is the predominant isoflavone in soybeans (Glycine max) and is also found in other legumes including chickpeas, kudzu, and alfalfa. It is classified as a phytoestrogen owing to its structural similarity to 17beta-estradiol and its capacity to bind estrogen receptors (ERalpha and ERbeta), with approximately 7- to 30-fold selectivity for ERbeta over ERalpha. Beyond its estrogenic activity, genistein possesses a range of pharmacological properties: it is a broad-spectrum tyrosine kinase inhibitor (IC50 approximately 10–100 μM for multiple kinases, competing with ATP at the catalytic site), a partial PPARgamma agonist, an epigenetic modulator (affecting DNA methylation and histone modifications), and a direct free-radical scavenger by virtue of its phenolic hydroxyl groups (5-OH, 7-OH, 4′-OH). These pleiotropic properties have positioned genistein as a candidate multi-target agent for IRI, where simultaneous engagement of oxidative, inflammatory, and cell-death pathways may offer advantages over single-target interventions. We hypothesize that genistein’s efficacy against IRI derives from its ability to synchronize redox homeostasis (via Nrf2), epigenetic regulation (via SIRT1), and immunomodulation (via microglia polarization), distinguishing genistein from synthetic antioxidants.

The aim of this review was to comprehensively analyze and critically synthesize the regulatory mechanisms of genistein and its specific role in ischemia-reperfusion injury across organ systems, with emphasis on: (1) distinguishing mechanistic evidence strength according to the level of causal validation, (2) highlighting pharmacokinetic and formulation barriers to clinical translation, (3) addressing assay-interference and PAINS (pan-assay interference compounds) liabilities relevant to polyphenolic natural products, (4) discussing drug-drug interaction risks relevant to IRI clinical contexts, and (5) proposing a prioritized translational roadmap.

## 2 Literature search methodology

This review employed a systematic literature search strategy. The search covered the period up to 31 December 2025, and included the following databases: PubMed/MEDLINE, Web of Science Core Collection, and Scopus. The search strategy combined MeSH terms and free-text keywords: (“genistein” OR “4′,5,7-trihydroxyisoflavone” OR “phytoestrogen”) AND (“ischemia-reperfusion injury” OR “reperfusion injury” OR “ischemic injury” OR “myocardial infarction” OR “cerebral ischemia” OR “stroke” OR “acute kidney injury” OR “renal ischemia” OR “hepatic ischemia” OR “liver ischemia” OR “intestinal ischemia” OR “testicular torsion” OR “retinal ischemia”).

Inclusion criteria were: (1) original research articles (*in vitro*, *in vivo*, or *ex vivo*); (2) administration of genistein or structurally characterized genistein derivatives (e.g., genistein-3′-sodium sulfonate, 7-difluoromethyl-5,4′-dimethoxygenistein); (3) use of an established IRI model with defined ischemia and reperfusion durations; and (4) reporting of at least one mechanistic endpoint (e.g., protein expression, enzyme activity, genetic manipulation, or pharmacological inhibitor studies). Exclusion criteria were: (1) studies using only complex soy extracts, mixed isoflavone preparations, or other isoflavones (e.g., biochanin A, daidzein) without isolated genistein data; (2) reviews, commentaries, editorials, and conference abstracts; (3) studies without IRI or hypoxia-reoxygenation models; (4) non-English language articles without available translations.

After duplicate removal, titles and abstracts were screened by two independent reviewers, with disagreements resolved by consensus. Full texts of potentially eligible studies were retrieved and assessed against inclusion criteria. Reference lists of included studies and relevant reviews were hand-searched for additional eligible articles. A total of approximately 80 studies were retained for qualitative synthesis. Data extracted from each study included species/strain, sex, age, organ model, ischemia-reperfusion protocol, genistein dose, route, timing relative to ischemia/reperfusion, vehicle, comparator, primary endpoints, mechanistic assays, and whether causal validation was performed (inhibitor, knockdown, knockout, or rescue experiments). Evidence was graded as: Level A = pathway involvement confirmed by genetic deletion, siRNA knockdown, or pharmacological inhibitor with rescue; Level B = pathway involvement supported by multiple protein/biochemical endpoints with consistent dose-response; Level C = inferred from protein expression changes alone without orthogonal validation.

## 3 Overview of genistein

### 3.1 Chemical properties and pharmacokinetics

**FIGURE 1:**
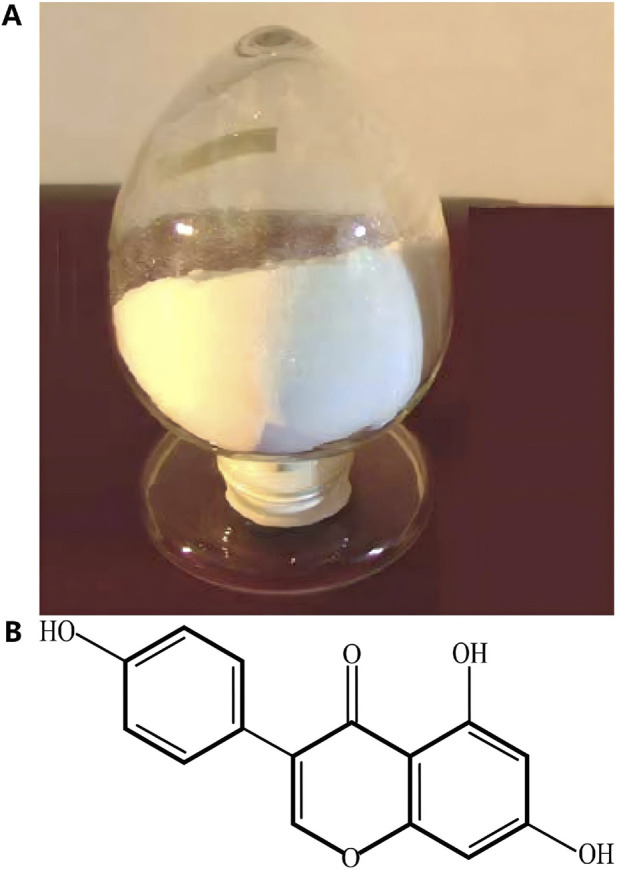
Morphology and molecular structure of Genistein. **(A)** shows the morphology of genistein; **(B)** shows the molecular structure of genistein.

Genistein is an off-white to pale-yellow crystalline powder (Figure 1A) and has a melting point ranging from 297 °C to 298 °C. The compound is soluble in dimethyl sulfoxide (DMSO) and ethanol, but has very low aqueous solubility (approximately 8.7 μg/mL, or approximately 32 μM, at 25 °C), with solubility largely independent of pH within the physiological range (Zheng et al., 2024; Chirumbolo, 2015). The isoflavone content in various types of plants may show differences depending on the plant species, cultivar, growing environment, and processing methods; soybean is generally considered one of the richest dietary sources, although quantitative claims must account for substantial variability across species, cultivars, and analytical methods (Butkute et al., 2014).

The pharmacokinetic profile of genistein presents substantial challenges for translational interpretation. Oral genistein, whether as purified compound or as soy isoflavone extract, undergoes extensive first-pass metabolism in the intestinal epithelium and liver. Absolute oral bioavailability is low (estimated less than 5%–15% in rodents and humans) due to extensive phase II conjugation (Setchell et al., 2001). Peak plasma concentrations (Cmax) of total genistein (aglycone plus conjugates) in humans following a 50 mg oral dose are approximately 0.5–2.5 μM, but free aglycone concentrations are typically 10- to 100-fold lower (approximately 10–50 nM), as greater than 95% of circulating genistein is present as glucuronide and sulfate conjugates (Setchell et al., 2001).

Genistein is an excellent substrate for UDP-glucuronosyltransferases (UGTs, particularly UGT1A1, UGT1A8, UGT1A9, and UGT1A10) and sulfotransferases (SULTs, particularly SULT1A1 and SULT1E1). Glucuronidation occurs predominantly at the 7-OH position, with minor conjugation at the 4′-OH position. These conjugates are efficiently excreted into bile (via MRP2/ABCC2 and BCRP/ABCG2) and urine (via organic anion transporters, OATs, and organic anion transporting polypeptides, OATPs). Enterohepatic recirculation prolongs the apparent half-life of total genistein to approximately 6–10 h in humans (Yang et al., 2012).

Plasma protein binding is extensive (primarily to albumin), with free fractions estimated at less than 1%–5%. Additional binding to sex hormone-binding globulin (SHBG) provides a secondary distribution compartment, although genistein’s affinity for SHBG is lower than that of endogenous estradiol. Tissue-to-plasma ratios vary by organ, with preferential accumulation reported in reproductive tissues, thyroid, and liver. Brain penetration is limited by the blood-brain barrier (BBB), with brain-to-plasma ratios typically less than 0.1 for the aglycone, although conjugated metabolites may enter via organic anion transporters.

A critical translational concern is the concentration disconnect between *in vitro* and *in vivo* studies. Most *in vitro* genistein studies employ concentrations of 10–100 μM, far exceeding achievable free plasma aglycone concentrations in humans or experimental animals. While local tissue concentrations or intracellular accumulation may exceed plasma levels, this has rarely been measured. Almost none of the reviewed preclinical IRI studies measured plasma or tissue genistein concentrations. When concentrations were reported, they typically reflected total genistein after enzymatic deconjugation rather than free aglycone. This represents a major evidence gap in the IRI literature (Table 1).

**TABLE 1:** Pharmacokinetic and formulation barriers.

Parameter	Current status in genistein IRI literature	Barrier to translation
Aqueous solubility	Approximately 32 μM; pH-independent	Limits i.v. formulation; precipitation risk
Oral bioavailability	Less than 5%–15% absolute; extensive first-pass metabolism	Low systemic exposure; high inter-individual variability
Free plasma aglycone	Less than 0.1 μM (dietary); up to 1–5 μM (high-dose supplement)	Orders of magnitude below *in vitro* effective concentrations (10–100 μM)
Phase II metabolism	Greater than 95% glucuronidated/sulfated; UGT1A1, SULT1A1 major enzymes	Rapid clearance; conjugate pharmacological activity unknown
Plasma protein binding	Greater than 95% bound (albumin, SHBG)	Low free fraction limits target engagement
Tissue penetration (BBB)	Brain:plasma ratio less than 0.1 for aglycone	Limited CNS target engagement
Vehicle effects	DMSO (free-radical scavenger), corn oil, CMC suspensions	Vehicle may confound IRI endpoints
In vitro-in vivo disconnect	10–100 μM *in vitro* vs. less than 0.1 μM free aglycone *in vivo*	*In vitro* mechanisms may not translate *in vivo*
Formulation for injection	No approved parenteral formulation exists	Cannot be administered i.v. in acute IRI settings
Clinical PK in IRI contexts	No data available	Unknown exposure in target patient population

### 3.2 Genistein’s pharmacological profile

Genistein contains a unique chemical structure consisting of multiple phenolic hydroxyl groups (Figure 1B). This special structure gives it distinctive pharmacological and biological activities (Bansal et al., 2024; Jaiswal et al., 2018). Studies have shown that genistein possesses antioxidant effects capable of effectively scavenging free radicals (superoxide anion, hydroxyl radical, peroxyl radicals, and peroxynitrite in cell-free assays) and reducing oxidative stress, thus playing a protective role in ischemia-reperfusion injury (Susanikova et al., 2019). In addition, genistein has been found to modulate inflammatory responses and influence apoptotic signalling pathways (Guo et al., 2019; Ji et al., 2004); these mechanisms of action together constitute the protective effect of genistein against ischemia-reperfusion injury.

It is important to recognize that genistein’s phenolic hydroxyl groups also confer pan-assay interference (PAINS) liability. The 4′-OH group can undergo one-electron oxidation to form phenoxyl radicals, capable of reducing tetrazolium salts (MTT, XTT, WST-1) and dichlorofluorescein (DCFH2-DA) independently of cellular effects. The 5-OH and 4-keto groups form a bidentate metal-chelating motif that can bind Fe2+/Fe3+, Cu2+, and Zn2+, non-specifically inhibiting metalloenzymes. Genistein also exhibits intrinsic fluorescence (excitation approximately 340 nm, emission approximately 480 nm), which can interfere with DCFH2-DA, MitoSOX, Fluo-4, JC-1, Annexin V-FITC, and TUNEL assays. At concentrations exceeding 50 μM, genistein can form colloidal aggregates in aqueous solution that non-specifically inhibit enzymes; this aggregation is reversible by detergent (0.01% Triton X-100 or Tween-20), providing a diagnostic test that is almost never performed in genistein IRI studies. These PAINS-related liabilities should be carefully considered when interpreting the genistein IRI literature (see detailed discussion in Section 9).

### 3.3 Solubility and formulation challenges

The low aqueous solubility of genistein imposes practical constraints on both experimental design and clinical development. In cell culture studies, DMSO is used as a vehicle at 0.05%–0.1% v/v; higher DMSO concentrations (greater than 0.5%) may independently affect cell viability, mitochondrial function, and kinase activities, complicating interpretation. In animal studies, genistein is typically administered intraperitoneally (i.p.) in DMSO, corn oil, or carboxymethylcellulose (CMC) suspensions. Each vehicle introduces potential confounders: DMSO is itself a free-radical scavenger and can alter membrane permeability; corn oil induces mild metabolic stress; CMC suspensions may exhibit variable dissolution and absorption kinetics.

To overcome these solubility limitations, several formulation strategies have been explored. Nanoparticle encapsulation using PLGA, liposomal, and solid-lipid nanoparticle formulations can enhance aqueous dispersibility and reportedly improve oral bioavailability by 2- to 5-fold in rodent models, though batch-to-batch variability, long-term stability, sterilization (for injectable formulations), and endotoxin control remain under-addressed. Phospholipid complexes (phytosomes) improve gastrointestinal absorption. Cyclodextrin inclusion complexes (hydroxypropyl-beta-cyclodextrin) increase apparent solubility but raise concerns about cholesterol extraction and hemolysis at high concentrations. Self-microemulsifying drug delivery systems (SMEDDS) can enhance lymphatic absorption and bypass first-pass metabolism.

Genistein-3′-sodium sulfonate (GSS) is a water-soluble sulfonated derivative designed for parenteral administration. GSS has been studied most extensively in cerebral IRI models (Xie et al., 2021; Jafari et al., 2023; Xie et al., 2023). The 3′-sulfonate substitution confers distinct pharmacokinetic advantages over the parent compound: (1) enhanced aqueous solubility (estimated greater than 50 mg/mL vs. approximately 0.0087 mg/mL for genistein), enabling intravenous bolus administration without organic co-solvents; (2) reduced plasma protein binding due to the anionic sulfonate group, potentially increasing the free fraction available for target engagement; (3) altered tissue distribution, with studies suggesting improved BBB penetration compared to genistein aglycone, attributed to organic anion transporter-mediated uptake; and (4) a distinct metabolic profile—the sulfonate moiety is resistant to phase II glucuronidation/sulfation at the modified position, potentially prolonging the circulation half-life of the pharmacologically active species. These properties enable GSS to achieve post-reperfusion therapeutic efficacy in cerebral IRI models (administered within 30 min to 2 h after reperfusion) (Xie et al., 2021; Xie et al., 2023; Li L. et al., 2017), a critical advantage over genistein which requires pretreatment.

However, GSS is a chemically modified entity whose receptor-binding profile, kinase inhibition spectrum, and off-target effects may differ from those of genistein. Head-to-head comparative pharmacokinetic and pharmacodynamic studies between genistein and GSS in the same IRI model have not been conducted. Furthermore, the long-term safety, immunogenicity, and metabolite profile of the sulfonate modification remain uncharacterized. GSS-specific evidence should be explicitly labeled as such and not conflated with genistein evidence without qualification.

Regarding clinical translation, no genistein formulation has been optimized specifically for IRI indications, and no human IRI clinical trials (phase I or beyond) have been conducted with genistein or GSS. However, oral genistein has been evaluated in several clinical contexts that provide relevant safety and pharmacodynamic data. In postmenopausal women, oral genistein (54 mg/day for 6–12 months) improved endothelial function (flow-mediated dilation), reduced markers of systemic inflammation (C-reactive protein, IL-6) (Villa et al., 2009; Squadrito et al., 2003).In postmenopausal women with metabolic syndrome, supplementation with pure genistein (54 mg/day for 12 months) significantly improves insulin resistance (HOMA-IR) and may have beneficial effects on left ventricular ejection fraction and left atrial function (De Gregorio et al., 2017). A phase II trial in prostate cancer patients demonstrated that oral genistein was well-tolerated at doses up to 300 mg/day for 6 months without dose-limiting toxicities (Fischer et al., 2004). While these studies were not conducted in IRI settings, they establish a preliminary safety and pharmacodynamic foundation: oral genistein at clinically administered doses can achieve plasma concentrations sufficient to modulate systemic inflammatory and oxidative biomarkers. The most plausible early-phase clinical contexts for IRI investigation remain elective surgical settings (renal transplantation, cardiac surgery) where prophylactic administration is feasible. At present, no genistein formulation has been optimized specifically for IRI indications, and head-to-head pharmacokinetic comparisons across formulation types in IRI models are lacking.

## 4 Effect of genisitein on ischemia-reperfusion injury

### 4.1 Effect of genistein on oxidative stress

Genistein, as a natural phytoestrogen of plant origin, has attracted attention in research for the treatment of ischemia-reperfusion injury due to its unique chemical structure that exhibits significant antioxidant capacity. In the pathophysiological process of ischemia-reperfusion injury, the overproduction of free radicals leads to lipid peroxidation as well as oxidative reactions of proteins and DNA, which further lead to impaired mitochondrial function and apoptosis, representing one of the key factors contributing to tissue damage. Genistein, by virtue of its unique chemical structure, is able to effectively scavenge free radicals, thereby reducing cellular damage caused by oxidative stress. Studies have shown that genistein has scavenging effects on various reactive oxygen radicals such as superoxide anion and hydroxyl radical, and possesses antioxidant activity that inhibits xanthine oxidase (Sato et al., 2011). However, it should be noted that the direct antioxidant activity of genistein as measured in cell-free systems (e.g., ORAC, DPPH, FRAP assays) is moderate compared to reference antioxidants such as quercetin, ascorbic acid, or Trolox. Furthermore, the concentrations required for meaningful direct radical scavenging (typically greater than 10–50 μM *in vitro*) substantially exceed the free plasma aglycone concentrations achievable through dietary or supplemental intake, questioning the *in vivo* relevance of direct antioxidant mechanisms.

Nrf2 (nuclear factor erythroid 2-related factor 2) is a crucial transcription factor responsible for regulating the gene expression of various antioxidant and detoxification enzymes, and HO-1 (heme oxygenase-1) is one of its downstream effector molecules. Under basal conditions, Nrf2 is sequestered in the cytoplasm by Keap1, which targets it for ubiquitin-proteasomal degradation. Studies have indicated that genistein, likely through its electrophilic phenolic oxidation products or through kinase-mediated Keap1 modification (involving PKC, PI3K, and/or ERK), disrupts the Keap1-Nrf2 interaction, allowing Nrf2 to translocate to the nucleus. Genistein was able to enhance the expression levels of enzymatic and non-enzymatic antioxidant genes and activate the production of HO-1 by promoting the expression of Nrf2, thereby reducing the generation of ROS, enhancing the antioxidant defence capacity of cells, and further attenuating the oxidative stress induced by IRI (Miao et al., 2018). In addition, genistein induces the transfer of Nrf2 from the cytoplasm to the nucleus, activating the antioxidant response element (ARE), which in turn initiates the expression of antioxidant enzyme genes and attenuates oxidative stress damage (Wang et al., 2013). Causal validation (Level A evidence) was provided by Nrf2 siRNA silencing in primary cortical neurons subjected to oxygen-glucose deprivation/reperfusion (OGD/R), which partially abrogated genistein’s protective effects in cerebral IRI (Wang et al., 2013). In renal and cardiac IRI models, genistein enhanced Nrf2 nuclear translocation and HO-1 expression, but causal validation via Nrf2 knockout or pharmacological inhibition in these organs has not been reported (Level B). In hepatic IRI, Nrf2/HO-1 involvement has been inferred from protein expression changes alone, without causal validation (Level C). The discovery of this mechanism provides a molecular-level explanation for the protective role of genistein in ischemia-reperfusion injury.

The ADORA2A receptor (adenosine A2A receptor) may reduce inflammation and oxidative stress through the activation of cyclic adenosine monophosphate (cAMP) and protein kinase (PK)-dependent pathways, broadly suppressing inflammation, reducing oxidative burst, and enhancing endothelial barrier function. It was demonstrated that genistein pretreatment was able to inhibit oxidative stress induced by IRI by upregulating ADORA2A expression (He et al., 2023). Importantly, co-administration of the selective ADORA2A antagonist SCH58261 abolished genistein’s renoprotective effects (including improvements in BUN, creatinine, tubular necrosis, and oxidative stress markers), establishing Level A evidence for this pathway in renal IRI. Whether ADORA2A upregulation occurs in other organs or represents a kidney-specific mechanism remains unknown.

In addition, the role of genistein in the regulation of oxidative stress is reflected in its maintenance of mitochondrial functional homeostasis (Rajput et al., 2017). Mitochondria, as the main site of free radical production in cells, also serve as key regulators of apoptosis. Genistein stabilises the mitochondrial membrane potential (Delta-psi-m) and reduces the opening of the mPTP, which in turn inhibits the release of cytochrome c and the translocation of apoptosis-inducing factor (AIF), thereby protecting cells from apoptosis (Fuchs et al., 2007). Some studies have suggested mitochondrial ATP-sensitive potassium channel (mKATP) involvement based on attenuation of genistein’s effects by the mKATP blocker 5-hydroxydecanoate (5-HD). However, evidence is complicated by: (1) the relatively non-specific pharmacology of 5-HD; (2) the possibility that genistein’s mitochondrial effects are indirect consequences of cytosolic signaling rather than direct mitochondrial actions; and (3) the fact that mitochondrial genistein concentrations after systemic administration have never been measured. Evidence level: B (cardiac, renal); C (other organs).

### 4.2 Anti-inflammatory effects of genistein

Experimental evidence indicates that genistein possesses anti-inflammatory pharmacological activity, and its mechanism of action is mainly manifested in the regulation of inflammatory mediators. It has been shown that pretreatment with genistein significantly reduces the levels of TNF-alpha and IL-6 in nerve cells, thereby reducing the inflammatory response and nerve damage (Li et al., 2022). Meanwhile, genistein has been found to inhibit the activation of nuclear factor-kappaB (NF-kappaB), an important transcription factor that regulates the expression of several inflammation-mediated genes. Genistein inhibits NF-kappaB activation through multiple mechanisms: stabilization of IkappaBalpha, preventing its phosphorylation-dependent degradation; inhibition of IKK (IkappaB kinase) activity, possibly through direct tyrosine kinase inhibition; enhanced SIRT1-mediated deacetylation of the p65/RelA subunit at Lys310; and competition with ERalpha-mediated co-activator recruitment to NF-kappaB-responsive promoters. By inhibiting the activation of NF-kappaB, genistein is able to reduce the production of inflammatory mediators and exert its anti-inflammatory effects at the molecular level (Li et al., 2022). However, it should be noted that NF-kappaB also has cytoprotective functions in some IRI contexts, including the induction of anti-apoptotic genes (cIAPs, Bcl-xL) and antioxidant enzymes (MnSOD, ferritin heavy chain). Therefore, genistein-mediated NF-kappaB inhibition may not be uniformly beneficial across all cell types and injury phases.

In addition, the NLRP3 inflammasome (NOD-, LRR- and pyrin domain-containing protein 3) has been found to play a key role in the inflammatory response to cerebral ischemia-reperfusion injury, and its activation aggravates the injury. The NLRP3 inflammasome is a multi-protein cytosolic complex that senses cellular stress signals (ROS, K+ efflux, lysosomal damage, mitochondrial DNA) and, upon activation, nucleates ASC (apoptosis-associated speck-like protein containing a CARD) to recruit and auto-activate caspase-1. Active caspase-1 cleaves pro-IL-1beta and pro-IL-18 into their mature secreted forms and cleaves gasdermin D (GSDMD), whose N-terminal fragment oligomerizes to form plasma membrane pores that mediate pyroptotic cell death. Genistein has been reported to reduce NLRP3, ASC, cleaved caspase-1, mature IL-1beta, and IL-18 protein levels in the ischemic hemisphere of reproductively senescent female mice subjected to transient middle cerebral artery occlusion (tMCAO), associated with reduced infarct volume and improved neurological scores (Wang et al., 2020). The mechanism was linked to neuronal G protein-coupled estrogen receptor (GPER/GPR30) signaling, as GPER antagonism with G15 partially reversed NLRP3 inhibition (Level B evidence). However, several mechanistic details remain unresolved: whether genistein affects NLRP3 inflammasome priming (signal 1: NF-kappaB-dependent NLRP3 and pro-IL-1beta transcription), assembly (signal 2: NLRP3-ASC nucleation), ASC speck formation, caspase-1 auto-activation, GSDMD cleavage, or IL-1beta/IL-18 maturation has not been systematically dissected. Furthermore, NLRP3 inhibition by genistein in organs other than the brain has not been demonstrated. This ability to modulate inflammatory mediators provides new perspectives for genistein in the clinical treatment of ischemia-reperfusion injury, especially under pathological conditions of inflammatory response.

Multiple signalling pathways are involved in the anti-inflammatory effects of genistein. The JAK/STAT signalling pathway has been shown to be involved in many biological processes, including cell proliferation, differentiation, apoptosis and tumour growth. In addition, it plays a key role in a number of inflammation-related diseases. It has been found that activation of the JAK2/STAT3 signalling pathway triggers the transcription and expression of inflammatory genes, such as TNF-alpha, IL-6 and IL-1beta, which in turn leads to excessive accumulation of inflammatory mediators. Genistein can demonstrate a protective effect against ischemia-reperfusion injury by inhibiting the JAK2/STAT3 signalling pathway (Li and Zhang, 2003). Genistein is hypothesized to inhibit STAT3 phosphorylation (Tyr705) through direct competition for the ATP-binding pocket of JAK2 (consistent with genistein’s broad kinase inhibitory activity) or through upstream inhibition of Src-family kinases. However, STAT3 also has well-established pro-survival functions in cardiomyocytes and neurons, where it drives the expression of anti-apoptotic Bcl-2 family members, antioxidant enzymes (MnSOD), and pro-angiogenic factors (VEGF). Consequently, STAT3 inhibition by genistein may be protective in inflammatory cell compartments (microglia, infiltrating macrophages) but potentially detrimental in parenchymal cells. The context- and cell-type-specificity of JAK2/STAT3 modulation by genistein has not been adequately addressed.

Meanwhile, genistein can also downregulate microglia M1 polarisation via the alpha7nAChR-NF-kappaB pathway, thereby improving neuroinflammation in a rat model of stroke (Xie et al., 2021). The alpha7 nicotinic acetylcholine receptor (alpha7nAChR) is a ligand-gated ion channel expressed on microglia, macrophages, and other immune cells, whose activation suppresses NF-kappaB signaling through the “cholinergic anti-inflammatory pathway.” Studies using genistein-3′-sodium sulfonate (GSS) in rat models of cerebral IRI demonstrated that GSS promoted microglial M2 polarization (increased Arg-1, IL-10, CD206; decreased iNOS, CD86) and reduced neuroinflammation through an alpha7nAChR-dependent mechanism, as the selective alpha7nAChR antagonist methyllycaconitine (MLA) abrogated these effects (Xie et al., 2021). This is Level A evidence for GSS but does not directly translate to genistein itself, and whether genistein directly binds alpha7nAChR, modulates receptor expression, or acts through cholinergic pathway components is unclear (Table 2).

**TABLE 2:** Organ-specific anti-inflammatory mechanisms of genistein in IRI.

Organ	Key inflammatory cell types	Genistein effect	Mechanism(s)	Evidence level
Cerebral	Microglia (M1/M2), astrocytes, monocytes/neutrophils	Reduced M1 polarization, NLRP3, TNF-alpha, IL-6	alpha7nAChR-NF-kappaB, NLRP3, JAK2/STAT3	A (alpha7nAChR with GSS); B (NLRP3, NF-kappaB)
Cardiac	Reduced neutrophil infiltration, TNF-alpha, ICAM-1	Cardiomyocytes, coronary endothelium, neutrophils	NF-kappaB, tyrosine kinase inhibition	B
Renal	Proximal tubular epithelium, macrophages, dendritic cells	Reduced tubular injury, macrophage infiltration	NF-kappaB, ADORA2A, SIRT1	A (ADORA2A); B (NF-kappaB, SIRT1)
Hepatic	Kupffer cells, LSECs, hepatocytes, neutrophils	Sparse data	Not well characterized	C (all)
Intestinal	Epithelial cells, Paneth cells, lamina propria macrophages Reduced	morphological injury, MPO	Not well characterized	C (all)

LSECs, liver sinusoidal endothelial cells; MPO, myeloperoxidase.

Genistein and Pyroptosis: Pyroptosis is a gasdermin-mediated, pro-inflammatory programmed cell death initiated by inflammasome activation (canonical: caspase-1; non-canonical: caspase-4/5/11) that has been increasingly recognized (2023–2025) as a major contributor to IRI pathology across organs. Emerging evidence suggests that genistein may suppress pyroptosis through the NLRP3/caspase-1/GSDMD axis. In cerebral IRI, genistein reduced cleaved caspase-1, GSDMD-N (the pore-forming N-terminal fragment), IL-1beta, and IL-18 levels (Wang et al., 2020). A seminal 2025 study by Yu et al. (2025) demonstrated that genistein-3′-sodium sulfonate (GSS) directly suppresses NLRP3-mediated cell pyroptosis after cerebral ischemia, providing the most direct evidence to date linking genistein derivatives to pyroptotic cell death inhibition in IRI contexts. In myocardial IRI models, recent studies (2024–2025) have indicated that genistein-mediated NLRP3 inflammasome inhibition attenuates cardiomyocyte pyroptosis, reducing lactate dehydrogenase (LDH) release and preserving membrane integrity. Furthermore, genistein has been shown to alleviate doxorubicin-induced cardiomyocyte autophagy and apoptosis via the ERK/STAT3/c-Myc signaling pathway (Wu et al., 2024), and improves heart failure with preserved ejection fraction through BNIP3L-mediated mitophagy (Dou et al., 2026), mechanisms that may extend to IRI cardioprotection. In renal IRI, pyroptosis of tubular epithelial cells contributes to AKI-to-CKD transition through sustained inflammatory signaling and tubulointerstitial fibrosis; however, genistein’s effects on renal pyroptosis markers (GSDMD cleavage, IL-1beta release, ASC speck formation) have not been specifically investigated beyond general NLRP3 endpoints. Key research gaps include: whether genistein directly binds inflammasome components (NLRP3, ASC, caspase-1) or acts indirectly through ROS reduction and mitochondrial stabilization; whether genistein modulates non-canonical pyroptosis (caspase-11/4/5); and whether pyroptosis inhibition contributes to genistein’s protective effects in organs beyond the brain and heart.

Genistein and Ferroptosis: Ferroptosis, an iron-dependent, lipid peroxidation-driven form of regulated necrosis characterized by glutathione depletion, GPX4 inactivation, and accumulation of lipid hydroperoxides, has emerged as a central mediator of IRI in the kidney, heart, brain, and liver (Zhang et al., 2026; Liu et al., 2023; Zhang et al., 2023; Song et al., 2026; Liu et al., 2025). Genistein possesses several physicochemical and pharmacological properties that theoretically position it as a ferroptosis modulator. Its 5-OH/4-keto chelating motif can bind Fe2+, reducing the labile iron pool and Fenton reaction-mediated lipid peroxidation (Boadi et al., 2003; Mira et al., 2002). Nrf2/HO-1 pathway activation transcriptionally upregulates multiple anti-ferroptotic defenses: GPX4 (the major phospholipid hydroperoxidase), glutathione biosynthetic enzymes (GCL, GSS), ferroportin (iron export), ferritin heavy chain (FtH, iron sequestration), and xCT (SLC7A11, the cystine/glutamate antiporter subunit of system Xc-that imports cystine for glutathione synthesis) (Zhang et al., 2025; Ding et al., 2023). However, direct experimental evidence for genistein modulation of ferroptosis in IRI models is exceedingly sparse. As of 2025, no study has systematically measured ferroptosis markers (GPX4 protein/activity, ACSL4, PTGS2/COX-2 mRNA, 4-HNE adducts, MDA, glutathione/GSSG ratio, labile iron pool, mitochondrial lipid peroxidation via C11-BODIPY or Liperfluo) in genistein-treated IRI models. This represents a critical underexplored dimension, particularly in renal IRI (where iron accumulation and ferroptosis are major drivers of proximal tubular injury) and cerebral IRI (where ferroptosis contributes to delayed neuronal death). Dedicated studies employing ferroptosis-specific inhibitors (ferrostatin-1, liproxstatin-1) as comparators and iron chelators (deferoxamine) as ortholog controls are needed to establish whether ferroptosis modulation contributes to genistein’s IRI-protective effects. Given the PAINS liability of genistein in iron/redox assays (see Section 6), such studies must include rigorous assay controls to distinguish genuine pharmacological effects from Fe2+ chelation artifacts.

The immune response is closely related to the inflammatory response and is considered to be one of the consequences of a strong immune response. Genistein is prominent in affecting a wide range of immune cells, including macrophages, T cells and B cells. Macrophages play a dual role in ischemia-reperfusion injury, and genistein can balance the immune response by modulating this role. Studies have shown that genistein is able to inhibit the role of M1-type macrophages in the inflammatory response by promoting their apoptosis and reducing the degree of inflammation (Cong et al., 2022). In contrast, genistein can also attenuate the inflammatory response by inhibiting macrophage overactivation and reducing the release of pro-inflammatory cytokines such as TNF-alpha and IL-6 (Deodato et al., 1999). Meanwhile, genistein was found to significantly reduce the inflammatory response induced by lipopolysaccharide (LPS), suggesting its potential clinical application in modulating immune cell functions (Huang et al., 2020). In addition, the T-cell regulatory effects of genistein cannot be excluded. They may affect the proliferation and differentiation of T cells that regulate the immune response. It has been found that genistein contributes to the maintenance of immune homeostasis and the reduction of tissue damage by inhibiting the differentiation of Th1 cells and promoting the activation of Th2 cells (Wang et al., 2008).

### 4.3 Effect of genistein on apoptosis

The protective effect of genistein against ischemia-reperfusion injury is partly due to the significant regulation of apoptosis-related proteins. Genistein may exert its anti-apoptotic effects by interfering with apoptotic signalling pathways through several different mechanisms. Studies have shown that genistein inhibits the expression of pro-apoptotic proteins (e.g., Bax and cleaved caspase-3) and increases the levels of anti-apoptotic proteins (e.g., Bcl-2) (Jafari et al., 2023; Li L. et al., 2017). However, it should be noted that in virtually all these studies, Bcl-2 family protein changes are reported as endpoint measurements without genetic or pharmacological validation that the Bcl-2/Bax axis is causally required for genistein-mediated protection. Bcl-2 family changes may therefore be markers of reduced cell death rather than mechanistic mediators; accordingly, Bcl-2/Bax evidence is graded as Level B across organs.

ERK1/2 proteins (extracellular signal-regulated kinase 1/2; p44/p42 MAPK) play an important role in cell proliferation and survival signalling pathways and belong to the MAPK family. Genistein has been found to activate the anti-apoptotic function of ERK1/2 by increasing its phosphorylation, contributing to the reduction of ischemia-reperfusion injury (Wang et al., 2014). However, it is essential to distinguish the three functionally distinct branches of the MAPK superfamily. ERK1/2 is classically a pro-survival and pro-proliferative pathway activated by growth factors (EGF, PDGF, IGF) through Ras-Raf-MEK-ERK signaling. In IRI, sustained ERK1/2 activation during reperfusion has been associated with anti-apoptotic effects via Bad phosphorylation and CREB-mediated Bcl-2 transcription. In contrast, JNK (c-Jun N-terminal kinase) is strongly activated by oxidative stress and is predominantly pro-apoptotic through phosphorylation of c-Jun, Bim/Bax activation, and mitochondrial translocation. p38 MAPK has pleiotropic roles in IRI including pro-inflammatory cytokine production and endothelial barrier disruption. Genistein has been reported to enhance ERK1/2 phosphorylation while concurrently inhibiting JNK phosphorylation (consistent with its ROS-scavenging and upstream kinase-inhibitory activities) (Wang et al., 2014; Liccardo et al., 2024). Erk1/2 and JNK/p38 should not be treated as a single “MAPK signalling” entity without contextual distinction.

**FIGURE 2:**
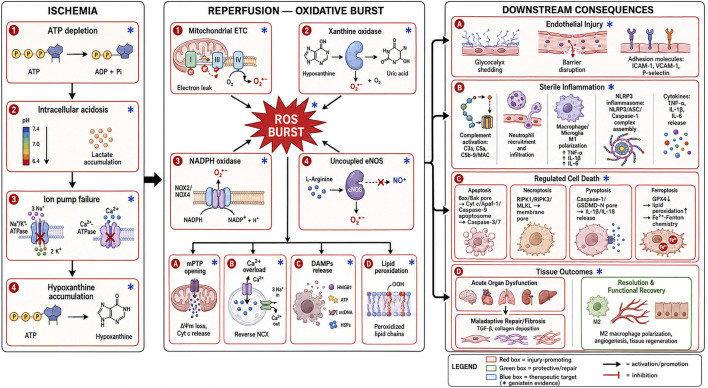
Integrated pathophysiology of ischemia-reperfusion injury. This schematic illustrates the convergent pathophysiological cascade of IRI across organs. The ischemic phase (left panel) depicts ATP depletion, intracellular acidosis (lactate accumulation), ion pump failure (Na+/K+-ATPase, Ca2+-ATPase), and accumulation of hypoxanthine (from ATP catabolism). The reperfusion phase (center panel) illustrates the oxidative burst: ROS generation from mitochondrial electron transport chain (Complex I/III), xanthine oxidase (converting accumulated hypoxanthine), NADPH oxidase (NOX2/NOX4), and uncoupled eNOS; mitochondrial permeability transition pore (mPTP) opening leading to loss of Delta-psi-m and cytochrome c release; calcium overload via reverse-mode Na+/Ca2+ exchanger (NCX); and release of DAMPs (HMGB1, ATP, mitochondrial DNA, HSPs). Downstream consequences (right panel) include: **(A)** endothelial injury (glycocalyx shedding, barrier disruption, adhesion molecule upregulation [ICAM-1, VCAM-1, P-selectin]); **(B)** sterile inflammation (complement activation [C3a, C5a, C5b-9/MAC], neutrophil recruitment and infiltration, macrophage/microglia M1 polarization, NLRP3 inflammasome assembly [NLRP3/ASC/caspase-1], and pro-inflammatory cytokine release [TNF-alpha, IL-1beta, IL-6]); **(C)** regulated cell death modalities: apoptosis (Bax/Bak mitochondrial pore, cytochrome c/Apaf-1/caspase-9 apoptosome, caspase-3/7 activation), necroptosis (RIPK1/RIPK3/MLKL phosphorylation and membrane pore formation), pyroptosis (caspase-1/GSDMD-N pore, IL-1beta/IL-18 release), ferroptosis (GPX4 inactivation, lipid peroxidation, iron-dependent Fenton chemistry); and **(D)** tissue outcomes: acute organ dysfunction, maladaptive repair/fibrosis (TGF-beta, collagen deposition), or resolution and functional recovery (M2 macrophage polarization, angiogenesis, tissue regeneration). Arrows indicate activation/promotion; T-bars indicate inhibition. Box colors: red = injury-promoting processes; green = protective/repair processes; blue = therapeutic intervention nodes (with genistein target sites indicated by asterisks where evidence exists). Source: Original figure based on referenced literature (Barrou et al., 2016; Zhang M. et al., 2024; Jafari et al., 2023; Villa et al., 2009; Squadrito et al., 2003; Wang et al., 2008; Liccardo et al., 2024; Gholampour et al., 2020; Zhang S. et al., 2024; Eckle et al., 2024; Xiang et al., 2024; Yinzhi et al., 2024; Hu et al., 2024).

Genistein has been found to be able to exert a protective effect through the synergistic action of apoptosis-related proteins. It has been shown that genistein upregulates the expression of SIRT1 (silent mating type information regulation 2 homolog 1, an NAD+-dependent class III histone deacetylase), which enhances the deacetylation of p53 protein (at Lys382), and at the same time, further promotes the degradation of p53 protein by genistein, which reduces apoptosis and protects against ischemia-reperfusion injury (Figure 2) (Li W. F. et al., 2017). Deacetylation of p53 by SIRT1 attenuates p53 transcriptional activity, reducing expression of pro-apoptotic targets (Bax, PUMA, Noxa) and p21-mediated cell-cycle arrest. In the renal IRI model by Li W. F. et al. (2017), genistein pretreatment (50 mg/kg, oral, 3 days) upregulated SIRT1 protein, enhanced p53 deacetylation, reduced acetyl-p53-mediated Bax transcription, and attenuated tubular apoptosis. Crucially, the SIRT1 inhibitor EX527 and SIRT1 siRNA both abrogated genistein’s renoprotective effects, establishing Level A evidence for the SIRT1/p53 axis in renal IRI. In cardiac IRI, SIRT1 involvement has been inferred but not causally validated (Level B).

The PI3K/Akt signalling pathway is a classical intracellular signalling pathway that plays a crucial role in cell proliferation, survival and metabolism. Studies have revealed that genistein binds to receptors on the cell membrane and activates PI3K, which in turn promotes the phosphorylation process of Akt (at Ser473). Phosphorylated Akt has the ability to inhibit apoptosis-associated proteins (e.g., Bad and Caspase-9), which can help to mitigate cellular damage induced by ischemia/reperfusion (Tissier et al., 2007; Gholampour et al., 2020). Through this mechanism, genistein has been shown to significantly reduce myocardial and renal damage (Tissier et al., 2007; Gholampour et al., 2020). In cardiac IRI, co-administration of the PI3K inhibitor LY294002 or wortmannin attenuated genistein’s cardioprotective effects, providing Level A evidence. In renal IRI, genistein-mediated Akt phosphorylation was linked to reduced tubular apoptosis and improved renal function; LY294002 co-treatment partially reversed protective effects, consistent with Level A evidence (Gholampour et al., 2020). In cerebral IRI, PI3K/Akt/mTOR pathway activation has been reported (Lu et al., 2019), though causal inhibitor studies are less definitive (Level B).

In addition, genistein also inhibited the activation of caspase family proteins, which play a key role in the execution of apoptosis. The results of the study showed that the apoptosis rate of genistein-treated cardiomyocytes was significantly lower than that of the untreated control group, suggesting that genistein has potential applications in cardioprotection (Ji et al., 2004). Genistein was also found to inhibit the activation of the JNK/p38 stress-responsive branches of the MAPK pathway by reducing the production of ROS, which in turn attenuates apoptosis (Liccardo et al., 2024). Through these mechanisms, genistein not only reduces cell death, but may also contribute to the maintenance of the structural and functional integrity of tissues, providing new perspectives for the treatment of ischemia-reperfusion injury (Table 3). However, caspase activation is a downstream executioner event common to many cell-death modalities, and its reduction does not provide specific mechanistic information about upstream signaling.

**TABLE 3:** Hierarchy of Genistein’s cytoprotective mechanisms in IRI.

Mechanism category	Target/Pathway	Evidence level by organ	Key caveats
Direct antioxidant (physicochemical)	Free-radical scavenging, Fe2+/Cu2+ chelation	C (all organs)	Requires >10 μM; unlikely at *in vivo* free concentrations
Estrogen receptor signaling (context-dependent)	ERalpha/ERbeta genomic and non-genomic	B (cerebral, cardiac); C (others)	Sex, age, endocrine status-dependent
Tyrosine kinase inhibition (broad, promiscuous)	Multiple RTKs and non-RTKs	C (all organs)	Low specificity; ATP-competitive
Nrf2/HO-1 (transcription-dependent)	Keap1-Nrf2-ARE-HO-1/NQO1/GCL/GST	A (cerebral); B (renal, cardiac); C (hepatic)	Validated by Nrf2 siRNA (cerebral)
SIRT1/p53 (NAD+-dependent deacetylation)	SIRT1-p53-p21-Bax axis	A (renal); B (cardiac)	Validated by EX527, SIRT1 siRNA (renal)
PI3K/Akt/mTOR (pro-survival)	PI3K-Akt-Bad/Caspase-9/GSK-3beta/eNOS	A (cardiac, renal); B (cerebral)	Validated by LY294002, wortmannin
NF-kappaB inhibition (secondary)	IkappaBalpha/NF-kappaB p65	B (cerebral, cardiac, renal)	NF-kappaB has dual pro-survival roles
JAK2/STAT3 (dual roles)	JAK2-STAT3 phosphorylation	B (cerebral); C (others)	STAT3 is pro-survival in parenchyma, pro-inflammatory in immune cells
alpha7nAChR (cell-type-specific)	alpha7nAChR-JAK2/STAT3-NF-kappaB	A (cerebral-microglia, with GSS)	Validated by MLA; GSS-specific; may not translate to genistein
ADORA2A-cAMP-PK (organ-specific)	ADORA2A-cAMP-PKA/PKC	A (renal)	Validated by SCH58261; unknown in other organs
NLRP3 inflammasome (immunomodulatory)	NLRP3-ASC-Caspase-1-IL-1beta/GSDMD	B (cerebral); C (others)	Validated by GPER/G15; incomplete mechanistic dissection
MAPK signaling (context-specific)	ERK1/2 (pro-survival) vs. JNK/p38 (stress)	B (cerebral, cardiac)	Net effect is cell-type-, concentration-, and context-specific
Mitochondrial protection (secondary)	mPTP, Delta-psi-m, cytochrome c, mKATP	B (cardiac, renal); C (others)	Direct vs. indirect effects unresolved

Evidence grading: A = genetic deletion/siRNA/pharmacological inhibitor with rescue; B = multiple protein/biochemical endpoints with dose-response; C = protein expression changes alone.

### 4.4 Cross-pathway interactions: SIRT1 as a mechanistic nexus

A critical insight emerging from this review is that genistein’s protective pathways do not operate in isolation but exhibit significant crosstalk, with SIRT1 serving as a central integrative node. Understanding these interactions is essential to move beyond a fragmented pathway inventory toward a coherent mechanistic model.

SIRT1-p53-NF-kappaB Axis: Genistein-mediated SIRT1 upregulation produces coordinated effects on two major transcription factors through deacetylation. First, SIRT1 deacetylates p53 at Lys382, attenuating p53 transcriptional activity and reducing expression of pro-apoptotic targets including Bax, PUMA, Noxa, and p21, thereby suppressing the intrinsic mitochondrial apoptotic pathway. Second, SIRT1 deacetylates the NF-kappaB p65/RelA subunit at Lys310, which reduces p65 DNA-binding affinity and transcriptional activity at pro-inflammatory gene promoters (TNF-alpha, IL-6, IL-1beta, ICAM-1, VCAM-1, iNOS, COX-2). This dual deacetylation mechanism enables genistein, via a single upstream target (SIRT1), to simultaneously suppress both apoptotic and inflammatory cascades—a mechanistic convergence that may explain observations in renal IRI where genistein concurrently reduces tubular apoptosis (via p53 deacetylation) and macrophage-driven inflammation (via NF-kappaB deacetylation) (Li W. F. et al., 2017).

NLRP3 Inflammasome and Pyroptosis Integration: The NLRP3 inflammasome requires two signals for full activation: Signal 1 (priming) involves NF-kappaB-dependent transcriptional upregulation of NLRP3 and pro-IL-1beta; Signal 2 (activation) involves assembly of the NLRP3-ASC-caspase-1 complex in response to cellular stress (ROS, K+ efflux, lysosomal damage). Genistein potentially suppresses NLRP3 at both levels. At the priming level, SIRT1-mediated NF-kappaB p65 deacetylation reduces NLRP3 and pro-IL-1beta transcription. At the activation level, genistein’s ROS-scavenging and mitochondrial-stabilizing effects (mPTP inhibition, Delta-psi-m preservation) may reduce the upstream stress signals that trigger NLRP3 assembly, while GPER/GPR30 signaling has been implicated in cerebral IRI (Wang et al., 2020). Activated caspase-1 subsequently cleaves gasdermin D (GSDMD) to generate the N-terminal pore-forming fragment that mediates pyroptotic cell death, and processes pro-IL-1beta/pro-IL-18 into mature cytokines. Thus, SIRT1-mediated NF-kappaB suppression and mitochondrial protection jointly converge on NLRP3 inflammasome regulation, linking oxidative stress sensing to inflammatory cell death.

Ferroptosis Connection: Ferroptosis is an iron-dependent, lipid peroxidation-driven form of regulated necrosis increasingly recognized as a major contributor to IRI, particularly in renal proximal tubular epithelium and cerebral neurons. Recent studies (Arzuk and Armagan, 2024; Mao et al., 2024) have begun exploring genistein’s potential anti-ferroptotic effects. Genistein’s 5-OH/4-keto bidentate motif enables Fe2+/Fe3+ chelation, which may reduce the labile iron pool available for Fenton chemistry. Concurrently, Nrf2/HO-1 activation upregulates glutathione synthesis (via GCL) and GPX4 expression, the central enzymatic defense against lipid peroxidation. Nrf2 also induces ferritin heavy chain (FtH) and ferroportin, promoting iron sequestration and export. However, direct evidence for genistein modulation of ferroptosis markers (GPX4, ACSL4, PTGS2, 4-HNE, MDA, labile iron pool) in IRI models is extremely limited. Only one or two studies have addressed ferroptosis endpoints, and systematic investigation across organs is absent. This represents a significant research opportunity, particularly in renal and cerebral IRI where ferroptosis is pathophysiologically prominent.

ADORA2A-cAMP-PK and PI3K/Akt Convergence: The ADORA2A-cAMP-PK and PI3K/Akt pathways converge on several downstream effectors. ADORA2A activation stimulates cAMP production, activating PKA and Epac, while PI3K/Akt phosphorylates eNOS (Ser1177), enhancing NO production and vasodilation. Both pathways converge on mitochondrial protection: Akt phosphorylates and inactivates GSK-3beta, reducing mPTP opening sensitivity, while PKA can phosphorylate and inhibit Drp1-mediated mitochondrial fission. Both pathways also inhibit pro-apoptotic Bad through phosphorylation at distinct sites, providing parallel anti-apoptotic inputs.

**FIGURE 3:**
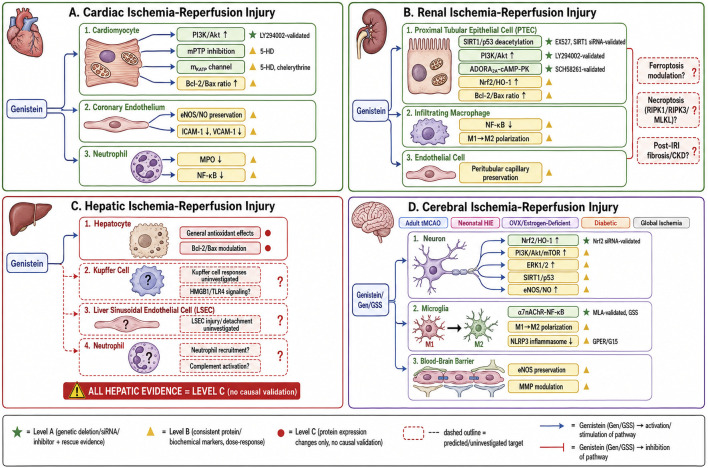
Organ-specific genistein mechanism map in ischemia-reperfusion injury. This multi-panel schematic maps genistein’s protective mechanisms to specific cell types and pathways across four organ systems with evidence-level indicators. **(A)** (Cardiac): Genistein targets cardiomyocytes (PI3K/Akt activation [LY294002-validated, Level A], mPTP inhibition, mKATP channel modulation [5-HD, Level B], Bcl-2/Bax ratio shift), coronary endothelium (eNOS/NO preservation, ICAM-1/VCAM-1 reduction), and infiltrating neutrophils (MPO reduction, NF-kappaB inhibition). **(B)** (Renal): Genistein targets proximal tubular epithelial cells (SIRT1/p53 deacetylation [EX527/siRNA-validated, Level A], PI3K/Akt [LY294002-validated, Level A], ADORA2A-cAMP-PK [SCH58261-validated, Level A], Nrf2/HO-1 [Level B]), infiltrating macrophages (NF-kappaB inhibition, M1-to-M2 polarization shift), and endothelial cells (peritubular capillary preservation). Ferroptosis and necroptosis pathways are indicated as uninvestigated (dashed). **(C)** (Hepatic): Genistein targets hepatocytes (general antioxidant effects, Bcl-2/Bax modulation [all Level C]); Kupffer cell and LSEC targets are predicted but uninvestigated (dashed outlines with question marks). HMGB1/TLR4 signaling, complement activation, and neutrophil recruitment are identified as research gaps. **(D)** (Cerebral): Genistein/GSS targets neurons (Nrf2/HO-1 [siRNA-validated, Level A], PI3K/Akt/mTOR [Level B], ERK1/2 activation [Level B], SIRT1/p53 [Level B]), microglia (alpha7nAChR-NF-kappaB [MLA-validated with GSS, Level A], M1-to-M2 polarization, NLRP3 inflammasome inhibition [GPER/G15, Level B]), and the BBB (eNOS preservation, MMP modulation). Adult stroke, neonatal HIE, OVX, and diabetic models are distinguished by separate sub-panel indicators. Evidence level symbols: green star (Level A), yellow triangle (Level B), red circle (Level C). Source: Original figure synthesizing data from referenced studies.

These cross-pathway interactions underscore that genistein’s protective effects are best understood not as an inventory of independently modulated targets, but as a coordinated, multi-level regulatory network centered on SIRT1-mediated transcriptional reprogramming, mitochondrial preservation, and inflammasome suppression. A high-level schematic integrating these pathways is presented in Figure 3.

## 5 Protective effects of genistein in different organs

Genistein, a naturally occurring isoflavone compound, has shown significant protective effects against ischemia-reperfusion injury through a number of different mechanisms. This compound possesses antioxidant, anti-inflammatory, and anti-apoptotic properties. However, the strength of mechanistic evidence and translational relevance varies substantially across organs and experimental paradigms.

### 5.1 Protective effects of genistein in cardiac ischemia-reperfusion injury

Cardiovascular disease is the leading cause of death worldwide (Yu W. et al., 2024). Myocardial ischemia is usually caused by coronary artery problems, and the goal of treatment is to restore blood flow quickly to reduce myocardial damage (Zhang S. et al., 2024). However, reperfusion may cause a more serious secondary injury, namely myocardial ischemia-reperfusion injury (Eckle et al., 2024), which has become a key factor in clinical outcomes.

Myocardial ischemia-reperfusion injury is associated with a variety of pathological mechanisms, including oxidative stress, mitochondrial dysfunction, calcium overload, endothelial glycocalyx shedding, neutrophil and platelet adhesion to activated endothelium, microvascular obstruction (“no-reflow” phenomenon), complement activation (C5b-9), and activation of regulated necrosis (necroptosis, ferroptosis) in addition to apoptosis (Xiang et al., 2024). Genistein, with its antioxidant and anti-inflammatory properties, has been shown to significantly improve cardiac function after the myocardium undergoes the ischemia-reperfusion process, thereby reducing the size of myocardial infarction by 20%–40% in rabbit and rat models of coronary artery occlusion-reperfusion. Its main mechanism of action lies in its ability to attenuate oxidative stress and inflammation by scavenging free radicals, reducing oxidative damage, and modulating the release of inflammatory mediators, while improving myocardial microcirculation, reducing microvascular permeability, and promoting cardiomyocyte recovery. At the same time, genistein can also inhibit apoptosis by directly regulating apoptosis-related proteins and pathways (e.g., caspase-3, Bcl-2/Bax ratio, PI3K/Akt), thus achieving the effect of cardioprotection (Ji et al., 2004; Li L. et al., 2017; Li W. F. et al., 2017).

Interestingly, during ischemia-reperfusion, myocardial tissue has been found to undergo an adaptive remodelling process, but excessive remodelling impairs myocardial function. Genistein is able to slow down the process of myocardial remodelling by modulating the composition of the extracellular matrix, allowing the myocardial structure to remain in a healthier state (Heusch, 2020). In addition, a clinical study in postmenopausal women reported that long-term genistein supplementation (54 mg/day, 6 months) improved endothelial function as assessed by flow-mediated dilation of the brachial artery (Squadrito et al., 2003). Further clinical trials have demonstrated that genistein supplementation in postmenopausal women improves cardiovascular risk factors including lipid profiles and insulin resistance (Crisaf et al., 2005), and these effects are preserved in women with metabolic syndrome (Irace et al., 2013). However, these studies were not IRI-specific trials, and the relevance of these findings to acute IRI protection in a broader patient population requires confirmation.

It must be emphasized that these data should be interpreted with caution. Most studies used acute pretreatment or peri-reperfusion administration in young, healthy, male animals. The relevance to the clinical scenario of ST-elevation myocardial infarction (STEMI), where ischemia time is unpredictable and patients are typically older with multiple cardiovascular risk factors (diabetes, hypertension, atherosclerosis), is limited. Furthermore, genistein’s effects on specific pathophysiological layers of myocardial IRI—such as endothelial glycocalyx injury, platelet activation, complement deposition, and the no-reflow phenomenon—have not been specifically investigated. Notably, a study by Colareda et al. (Colareda et al., 2020) demonstrated that mKATP channels and protein kinase C are involved in the cardioprotective effects of genistein on estrogen-deficient rat hearts exposed to ischemia/reperfusion through an energetic mechanism, providing mechanistic insight into sex-dependent cardioprotection. More recently, genistein has been shown to enhance the beneficial effects of exercise on antioxidant and anti-inflammatory balance and cardiomyopathy in ovariectomized diabetic rats (Mohammadi et al., 2025), a model more representative of the postmenopausal metabolic syndrome population at risk for IRI. These studies suggest that genistein can significantly ameliorate myocardial ischemia-reperfusion injury in preclinical models, but additional validation in large-animal models and comorbidity-rich settings is needed before clinical translation can be considered (Table 4).

**TABLE 4:** Evidence map for cardiac IRI.

Model	Species	Genistein Dose/Route/Timing	Key endpoints	Mechanism(s)	Causal validation	Evidence level	References
Rabbit LAD occlusion	Rabbit	1 mg/kg i.v., 10 min	pre-reperfusion Infarct size, CK-MB, caspase-3	Tyrosine kinase inhibition, anti-apoptotic	None specific	B	Ji et al. (2004)
Rat LAD occlusion	Rat	10 mg/kg i.p., pre-ischemia	Infarct size, Bcl-2/Bax, Akt-p	PI3K/Akt	LY294002	A	Tissier et al. (2007)
Rat Langendorff (OVX)	Rat (F)	0.5–2 mg/kg i.p., pretreatment	LVDP, mechanical efficiency	mKATP channels, PKC	5-HD, chelerythrine	B	Colareda et al. (2015)
Rat myofilament Ca2+ sensitivity	Rat	10–50 μM (*ex vivo* bath)	Ca2+ sensitivity, contractility	Myofilament modulation	None	C	Min et al. (2002)

LAD, left anterior descending coronary artery; CK-MB, creatine kinase-MB; OVX, ovariectomized; LVDP, left ventricular developed pressure.

### 5.2 Protective effects of genistein in hepatic ischemia-reperfusion injury

In liver tissue, ischemia-reperfusion injury is a complex process, which not only causes apoptosis, but also leads to significant impairment of liver function (Yinzhi et al., 2024). At the same time, ischemia-reperfusion injury also interferes with the normal metabolic function of the liver, affecting its ability to detoxify and synthesise proteins, thus further exacerbating the overall dysfunction of the liver (Hu et al., 2024).

Hepatic IRI has a unique pathophysiology involving distinct phases: early Kupffer cell (resident hepatic macrophage) activation and ROS production within minutes of reperfusion; liver sinusoidal endothelial cell (LSEC) injury and detachment, causing microcirculatory disruption; neutrophil recruitment via chemokine (CXCL1, CXCL2) and adhesion molecule (ICAM-1) upregulation; CD4^+^ T cell-mediated late-phase injury; mitochondrial oxidative stress and hepatocyte necrosis/apoptosis; HMGB1/TLR4-mediated sterile inflammatory amplification; and bile acid-related injury (Yu Q. et al., 2024).

The protective effect of genistein in the liver is demonstrated by its ability to enhance the antioxidant defence system within liver cells. When the liver undergoes a period of ischemia, i.e., insufficient blood supply, liver cells are subjected to severe stress and damage. Subsequently, when blood is reintroduced to the liver, i.e., reperfusion, the process that was intended to restore liver function instead exacerbates cellular damage. This reperfusion injury leads to the production of large amounts of ROS within the cells, triggering an inflammatory response and activation of apoptotic pathways, ultimately leading to liver cell death (Peng et al., 2022; Soares et al., 2019). Genistein, on the other hand, is able to reduce the damage to hepatocytes caused by oxidative stress. At the same time, genistein is able to inhibit the production of inflammatory mediators in the liver and reduce the inflammatory response of liver cells. This reduces the extent of liver damage and improves liver function. Genistein can also help repair damaged liver tissue by promoting the proliferation and regeneration of liver cells (Ak et al., 2019). This effect may be achieved by modulating signalling pathways associated with the cell cycle.

However, it must be acknowledged that the genistein literature for hepatic IRI is substantially less developed than for cardiac, renal, and cerebral IRI. Critical gaps include: (1) no study has specifically investigated Kupffer cell or LSEC responses to genistein in hepatic IRI; (2) neutrophil infiltration and HMGB1/TLR4 signalling have not been examined; (3) no causal validation via pathway inhibitors, genetic knockout, or cell-type-specific approaches exists—all hepatic IRI evidence is Level C (inferred from protein expression changes alone); (4) bile acid toxicity and cholangiocyte injury, which are important in liver IRI, have not been addressed; (5) whether genistein affects hepatocyte proliferation and liver regeneration after IRI, which is critical for recovery, is unknown. In conclusion, genistein, as a potential therapeutic drug for hepatic IRI, deserves further study, but substantially more primary research is required before mechanistic conclusions can be drawn.

### 5.3 Protective effects of genistein in renal ischemia-reperfusion injury

In clinical practice, compared with dialysis treatment, renal transplantation brings longer life expectancy as well as better quality of life to patients with end-stage renal disease. At the time of transplantation, renal ischemia-reperfusion injury may occur as a specific pathology during this process (Wu et al., 2025), with significant impact on the prognosis of renal transplant recipients (Reichardt et al., 2024; Burmakin et al., 2024).

Renal IRI involves coordinated injury to multiple nephron segments and cell types: proximal tubular epithelial cells (particularly the S3 segment) undergo cytoskeletal disruption, loss of polarity, and detachment; endothelial injury causes peritubular capillary rarefaction and impaired microvascular perfusion; dendritic cells and resident macrophages initiate sterile inflammation; infiltrating neutrophils and Ly6C-high monocytes amplify injury; complement alternative pathway activation (C3a, C5a, C5b-9) occurs; mitochondrial fragmentation (Drp1-mediated fission) and impaired mitophagy contribute; regulated necrosis including ferroptosis (particularly in PTECs) and necroptosis (RIPK1/RIPK3/MLKL) are activated; and maladaptive repair leads to interstitial fibrosis and progression to chronic kidney disease (CKD). Therefore, in-depth study and effective prevention of renal ischemia-reperfusion injury is of non-negligible importance.

Genistein has significant effects in reducing renal tissue damage, protecting renal cells from further damage through multiple mechanisms. Specifically, genistein can exert its protective effects on the kidneys through a variety of pathways, including antioxidant, anti-inflammatory, and modulation of apoptosis, thus providing a potential natural drug option for the treatment of renal ischemia-reperfusion injury in the clinical setting. Genistein was found to significantly improve the function of renal tubules in damaged kidneys, a type of injury often caused by a variety of external factors, including vascular surgery, acute impairment of the circulatory system, and blockage of the renal vascular network (Han et al., 2024).

**FIGURE 4:**
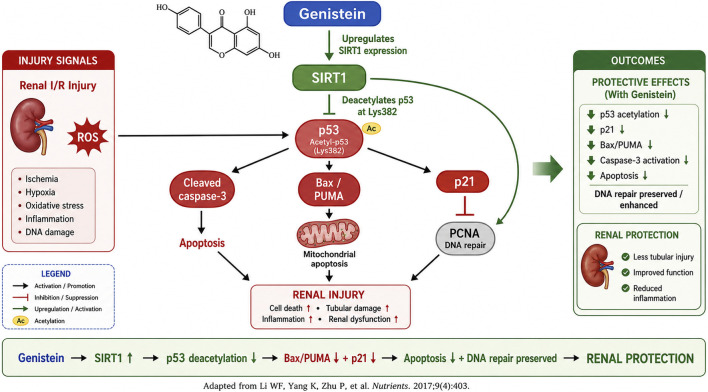
Translational decision pathway for genistein in IRI. This flowchart depicts the proposed translational development pathway. Tier 1 (Prophylaxis for Planned IRI Settings: highest feasibility, strongest rationale): Renal transplantation (genistein in preservation solutions + recipient pretreatment), elective cardiac surgery with cardiopulmonary bypass (preoperative genistein + cardioplegia additive), elective carotid/vascular surgery with planned temporary clamping, and planned high-risk PCI. Tier 2 (Peri-Reperfusion Intervention: moderate feasibility, requires parenteral formulation): GSS or nanoformulated genistein administered at reperfusion in controlled settings (transplant reperfusion, post-cardiac surgery). Tier 3 (Post-Injury Remodeling: lower feasibility, chronic oral administration): Long-term genistein for post-IRI fibrosis prevention (cardiac remodeling, renal CKD progression) and secondary prevention in high-risk populations. Derivative/nanoformulation development is indicated as a parallel track to improve bioavailability and enable parenteral administration. Prerequisites (gatekeeping checkpoints) at each stage: PK/PD characterization, large-animal efficacy and safety, orthogonal assay validation with PAINS controls, comorbidity-stratified studies, and formal drug-drug interaction profiling. Red stop signs indicate current evidence gaps that must be addressed before clinical investigation. Green arrows indicate pathways with the most supportive preclinical evidence. Source: Original figure.

Genistein scavenges free radicals in the body, reduces the level of oxidative stress, and attenuates renal tubular cell damage. This process involves regulating the expression of antioxidant enzymes such as superoxide dismutase (SOD) and glutathione peroxidase (GPx). At the same time, genistein was able to reduce serum levels of creatinine and urea nitrogen, both of which are commonly used to assess kidney function (Li W. F. et al., 2017; Gholampour et al., 2020; Ak et al., 2019). Importantly, the renal IRI literature for genistein is the most mechanistically rigorous among organ systems, with Level A evidence established for three distinct pathways: (1) SIRT1/p53 deacetylation-dependent anti-apoptotic signaling, validated by the SIRT1 inhibitor EX527 and SIRT1 siRNA (Figure 4) (Li W. F. et al., 2017); (2) ADORA2A-cAMP-PK pathway activation, validated by the selective ADORA2A antagonist SCH58261 (Wang et al., 2013); and (3) PI3K/Akt pro-survival signaling, validated by the PI3K inhibitor LY294002 (Gholampour et al., 2020).

Despite this mechanistic rigor, several gaps remain: (1) no study has examined whether genistein modulates ferroptosis or necroptosis in renal IRI, despite strong evidence that these regulated necrosis modalities contribute to AKI; (2) endothelial versus tubular cell primary targets have not been distinguished; (3) long-term outcomes—specifically the transition from AKI to fibrosis and CKD—have not been assessed; (4) large-animal models (porcine, non-human primate) are entirely absent; and (5) all studies used pretreatment paradigms; effects of post-reperfusion administration are unknown. These findings suggest that genistein has significant renoprotective potential and is expected to be a candidate renoprotective agent, though additional validation in clinically relevant models is warranted (Table 5).

**TABLE 5:** Evidence map for renal IRI.

Model	Genistein dose/Route/Timing	Key endpoints	Mechanism(s)	Causal validation	Evidence level	References
Rat bilateral renal IRI	50 mg/kg p.o., 3 days pretreatment	Cr, BUN, tubular necrosis, apoptosis	SIRT1/p53	EX527, SIRT1 siRNA	A	Li W. F. et al. (2017)
Rat renal IRI	10–40 mg/kg i.p., pretreatment	Cr, BUN, MDA, GSH, ADORA2A expression	ADORA2A-cAMP-PK	SCH58261	A	He et al. (2023)
Rat ischemic AKI	10 mg/kg i.p., 7 days pretreatment	Cr, BUN, histology, oxidative markers	PI3K/Akt	LY294002	A	Gholampour et al. (2020)
Rat adnexal torsion	Variable i.p., pre-detorsion	Ovarian histology, MDA, MPO	General antioxidant	None	C	Yazici et al. (2007)

Cr, serum creatinine; BUN, blood urea nitrogen; MDA, malondialdehyde; GSH, reduced glutathione; MPO, myeloperoxidase.

### 5.4 Protective effects of genistein in cerebral ischemia-reperfusion injury

Cerebral ischemia-reperfusion injury is a complex medical phenomenon, which refers to the fact that after the brain undergoes an ischemic phase, when the blood re-flows into the originally ischemic area, instead of bringing about the expected recovery, it triggers a more serious tissue damage process (Li et al., 2024). Therefore, in-depth exploration of the pathophysiological mechanisms of cerebral ischemia-reperfusion injury and searching for effective preventive and intervention strategies are of great significance in improving the clinical outcomes of patients with cerebrovascular diseases.

Cerebral IRI is the primary pathology underlying ischemic stroke, global cerebral ischemia (e.g., cardiac arrest), and neonatal hypoxic-ischemic encephalopathy (HIE). These are biologically and clinically distinct conditions that should not be conflated. The pathophysiology involves excitotoxicity (glutamate-induced Ca2+ overload via NMDA/AMPA receptors), oxidative/nitrosative stress (mitochondrial ROS, NADPH oxidase, nNOS/iNOS-derived NO/peroxynitrite), BBB disruption and vasogenic edema, neuroinflammation (microglial activation, astrogliosis, neutrophil and monocyte infiltration), cortical spreading depolarizations, and delayed neuronal death in selectively vulnerable regions (hippocampal CA1, cortical layers 3/5, striatal medium spiny neurons) (Chen et al., 2024; Zhang Y. et al., 2024; Cai et al., 2024).

In the course of intensive research on ischemic brain injury, genistein has been found to have protective potential for neurons. Genistein has apoptosis-inhibiting properties, which can further protect neurons from further damage by preventing programmed cell death, while restoring cognitive deficits in the brain. Genistein also exerts its protective effects through a variety of other mechanisms: specifically, it significantly reduces neuroinflammation, which lessens the inflammatory response in damaged areas of the brain, and it reduces oxidative damage due to its antioxidant properties that effectively scavenge free radicals. In conclusion, these mechanisms work together to enable genistein to show potential in the treatment of ischemic brain injury (Xie et al., 2023; Wang et al., 2014; Lu et al., 2019).

Sex, age, and endocrine status considerations are particularly important for genistein in cerebral IRI given its phytoestrogenic activity. Adult male rodents, young females, ovariectomized females, aged reproductively senescent females, and neonates represent biologically distinct populations. Genistein may exert different effects depending on circulating estrogen levels, ERalpha/ERbeta expression ratios (which change with age and injury), and developmental stage. Furthermore, focal ischemia (tMCAO) versus global ischemia models differ substantially in pathophysiology and therapeutic responsiveness; these variables should not be merged without qualification.

Genistein’s neuroprotective effects have been evaluated in multiple rodent models. In adult tMCAO, genistein (10 mg/kg/day, i.p., 14-day pretreatment) reduced infarct volume, improved neurological deficit scores, and reduced brain edema in male and ovariectomized female rats (Miao et al., 2018; Wang et al., 2013; Wang et al., 2014; Lu et al., 2019). GSS administered immediately after reperfusion also reduced infarct volume and improved functional outcomes (Xie et al., 2021; Xie et al., 2023; Li L. et al., 2017), demonstrating post-treatment efficacy in some experimental settings, though the time window is narrow (within 30 min to 2 h). In global cerebral ischemia, genistein reduced hippocampal CA1 neuronal loss and improved spatial memory, linked to Nrf2/HO-1 and eNOS upregulation (Wang et al., 2013). In neonatal HIE (Rice-Vannucci model), GSS reduced brain infarct volume, attenuated neuronal apoptosis, and improved long-term neurobehavioral outcomes (Xie et al., 2023). In streptozotocin-induced diabetic mice subjected to global cerebral ischemia, genistein attenuated neurological deficits, associated with DPP-4 inhibition (Rajput et al., 2017); this represents the only genistein IRI study conducted in a comorbid model.

Providing new ideas and possibilities for future clinical treatments, these studies collectively indicate that genistein—and its more water-soluble derivative GSS—exerts multi-mechanism neuroprotection in cerebral IRI. However, the predominance of pretreatment paradigms, the reliance on rodent models, and the limited comorbidity data constrain the translational interpretation of these findings (Table 6).

**TABLE 6:** Evidence map for cerebral IRI.

Model	Species/Sex/Age	Compound/Dose/Route/Timing	Key endpoints	Mechanism (s)	Causal Validation	Evidence Level	References
tMCAO	Rat/OVX F/adult	Gen 10 mg/kg i.p., 14 days pre	Infarct volume, Nrf2, HO-1, SOD	Nrf2/HO-1	Nrf2 siRNA (partial)	A	Miao et al. (2018), Wang et al. (2013)
tMCAO	Mouse/reprod. senesc. F	Gen 10 mg/kg i.p., 3 days pre	Infarct, NLRP3, IL-1beta, GPER	NLRP3 inflammasome, GPER	GPER antagonist G15	B	Wang et al. (2020)
tMCAO	Rat/M/adult	GSS 15–30 mg/kg i.v., post - reperfusion	Infarct, neuroscore, alpha7nAChR	alpha7nAChR - JAK2/STAT3	MLA	A (GSS)	Xie et al. (2021)
tMCAO	Mouse/OVX F	Gen 10 mg/kg i.p., 7 days pre	Infarct, ERK - p, caspase - 3	ERK1/2	None	B	Wang et al. (2014)
Global cerebral ischemia	Rat/M	Gen 10 mg/kg i.p., 7 days pre	CA1 neuronal survival, memory	Nrf2, eNOS	None	B	Wang et al. (2013)
Neonatal HIE (Rice-Vannucci)	Rat/P7 neonates	GSS 10–30 mg/kg i.p., post-HI	Infarct, neurobehavior	PI3K/Akt/mTOR, JAK2/STAT3	None	B (GSS)	Xie et al. (2023)
Global ischemia + diabetes	Mouse/M (STZ)	Gen 2.5–10 mg/kg i.p., pre	Neurological score, DPP-4	Nrf2, mitochondrial	None	C	Rajput et al. (2017)

OVX, ovariectomized; STZ, streptozotocin; P7, postnatal day 7; tMCAO, transient middle cerebral artery occlusion; HIE, hypoxic-ischemic encephalopathy.

### 5.5 Other organs

Intestinal IRI: In rat superior mesenteric artery occlusion-reperfusion models, genistein reduced morphological injury (Chiu score), myeloperoxidase (MPO) activity, and lipid peroxidation (Sato et al., 2011). Evidence is limited to one or two studies with correlative endpoints (Level C). Testicular IRI: In rat testicular torsion-detorsion models, genistein reduced oxidative stress, preserved spermatogenesis, and modulated the Notch2/Jagged1/Hes1 and MMP/TIMP systems (Al-Maghrebi and Renno, 2016) (Level B). Retinal IRI: Genistein, administered as a tyrosine kinase inhibitor, reduced retinal ganglion cell loss and preserved retinal thickness after intraocular pressure-induced retinal IRI in rats (Hayashi et al., 1997) (Level C). Gastric IRI: Genistein postconditioning via the capsaicin receptor (TRPV1) reduced gastric IRI in rats (Du et al., 2010); genistein postconditioning also protected human gastric epithelial cells against hypoxia/reoxygenation injury (Li et al., 2009) (Level B).

### 5.6 Summary of organ-specific dosing and mechanisms

To facilitate cross-organ comparison of genistein’s protective effects, a consolidated summary of effective doses, primary mechanisms, and evidence levels is provided.

**FIGURE 5:**
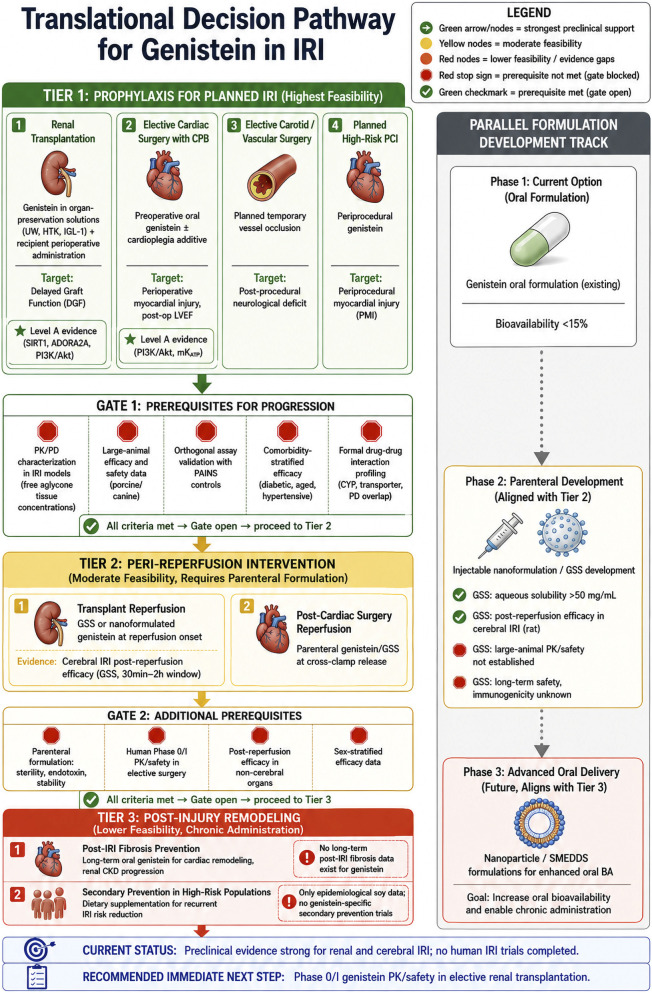
SIRT1/p53 mechanistic map of genistein in IRI-induced kidney injury (supplementary) (Li W. F. et al., 2017). Damage signalling causes p53 activation, which promotes expression of p21. p21 inhibits PCNA, a DNA repair factor, exacerbating cellular damage. Increased p53 also activates cleaved caspase-3, leading to apoptosis. Genistein promotes SIRT1 expression, which deacetylates p53 at Lys382, reducing pro-apoptotic transcription (Bax, PUMA), decreasing apoptosis, and protecting against IRI. Arrows = activation; T-bars = inhibition. Green = protective; red = injury-promoting.

Clinical Transformation Pathway (Figure 5): This flowchart depicts a tiered translational development pathway for genistein in IRI. Tier 1 (Prophylaxis in Planned IRI Settings: highest feasibility, strongest rationale) encompasses: (A) Renal transplantation—adding genistein to organ-preservation solutions (e.g., UW, HTK, IGL-1) plus recipient perioperative administration, targeting delayed graft function; (B) Elective cardiac surgery with cardiopulmonary bypass—preoperative oral genistein plus addition to cardioplegia solutions; (C) Elective carotid endarterectomy or intracranial aneurysm surgery with planned temporary vessel occlusion; (D) Planned high-risk percutaneous coronary intervention, targeting periprocedural myocardial injury. Tier 2 (Peri-Reperfusion Intervention: moderate feasibility) requires parenteral formulation development (GSS or nanoformulated genistein) and targets controlled reperfusion settings. Tier 3 (Post-Injury Remodeling: lower feasibility, chronic administration) includes long-term oral genistein for post-IRI fibrosis prevention (cardiac remodeling, renal CKD progression) and secondary prevention in high-risk populations. Prerequisites at each stage include: PK/PD characterization, large-animal safety and efficacy, orthogonal assay validation with PAINS controls, comorbidity-stratified efficacy studies, and formal drug-drug interaction profiling. Red stop signs indicate current evidence gaps; green arrows indicate pathways with the most supportive preclinical evidence (Table 7).

**TABLE 7:** Consolidated summary of genistein dosing and mechanisms across organs.

Organ	Model	Compound	Effective dose range	Route	Timing	Primary mechanisms (Evidence Level A/B)	Highest evidence level
Heart	*In vivo* rat	Genistein	0.5–10 mg/kg	i.p	Pretreatment	PI3K/Akt (A), mKATP/PKC (B)	A (PI3K)
Kidney	*In vivo* rat	Genistein	10–50 mg/kg	p.o./i.p	Pretreatment	SIRT1/p53 (A), ADORA2A-cAMP (A), PI3K/Akt (A)	A (3 pathways)
Brain	*In vivo* rat	Genistein	10 mg/kg × 7–14 days	i.p	Pretreatment	Nrf2/HO-1 (A), ERK1/2 (B)	A (Nrf2)
Brain	*In vivo* rat	GSS	15–30 mg/kg	i.v	Post-reperfusion	alpha7nAChR (A), JAK2/STAT3 (B)	A (alpha7nAChR, GSS)
Liver	*In vivo* rat	Genistein	5–15 mg/kg	i.p	Pretreatment	General antioxidant (C)	C
Intestine	*In vivo* rat	Genistein	10 mg/kg	i.p	Pretreatment	General antioxidant (C)	C
Testis	*In vivo* rat	Genistein	Variable	i.p	Pretreatment	Notch2/Jagged1/Hes1 (B)	B

## 6 Pains and assay-interference considerations

A critical but often overlooked issue in the genistein IRI literature is the compound’s potential for pan-assay interference (PAINS) and non-specific assay readout. Genistein is a polyphenolic isoflavone with several structural features that confer assay-interference liability.

First, redox activity: the phenolic hydroxyl groups (particularly 4′-OH) can undergo one-electron oxidation to form phenoxyl radicals, which can reduce tetrazolium salts (MTT, XTT, WST-1), dichlorofluorescein (DCFH2-DA), and other redox-sensitive probes independently of cellular effects, generating false-positive “antioxidant” or “viability” signals. Second, metal chelation: the 5-OH and 4-keto groups form a bidentate metal-chelating motif capable of binding Fe2+/Fe3+, Cu2+, and Zn2+, which can inhibit metalloenzymes non-specifically and interfere with Fe2+-dependent ferroptosis assays, metal-catalyzed ROS generation, and metal-based detection reagents. Third, fluorescence interference: genistein exhibits intrinsic fluorescence (excitation approximately 340 nm, emission approximately 480 nm in aqueous media), which can interfere with fluorescence-based assays including DCFH2-DA, MitoSOX, Fluo-4, JC-1, Annexin V-FITC, and TUNEL if not properly controlled with compound-only blanks. Fourth, colloidal aggregation: at concentrations exceeding 50 μM, genistein can form colloidal aggregates in aqueous solution that non-specifically inhibit enzymes (including kinases, which is noteworthy given genistein’s known tyrosine kinase inhibitory activity). Aggregation is reversible by detergent (0.01% Triton X-100 or Tween-20), providing a diagnostic test that is almost never performed in genistein IRI studies. Fifth, broad kinase inhibition: genistein competes with ATP at the kinase catalytic site with IC50 values of 10–100 μM for dozens of kinases, making it difficult to attribute biological effects to specific kinase pathways. Sixth, fluorescence quenching: genistein can quench the fluorescence of protein-tryptophan residues and extrinsic fluorophores, potentially interfering with FRET-based assays, thermal shift assays, and fluorescence polarization measurements.

It is important to acknowledge that none of the reviewed genistein IRI studies explicitly addressed PAINS liabilities. This does not invalidate the existing evidence but introduces a level of uncertainty that should be transparently discussed. Recommendations for future genistein IRI research include: (1) all cell-based fluorescence and luminescence assays should include genistein-only and vehicle-only controls at every concentration tested; (2) key mechanistic findings should be confirmed with at least one orthogonal, non-fluorescence-based method (e.g., HPLC-based ROS quantification, Western blot, ELISA, electron microscopy); (3) detergent-sensitivity controls (0.01% Triton X-100) should be employed to exclude colloidal aggregation artifacts; (4) concentration-response studies should show mechanistic effects at concentrations less than 10 μM to increase confidence in pathway-specific rather than non-specific effects; and (5) negative-control compounds with similar physicochemical properties but lacking the specific biological activity of interest (e.g., daidzein, which lacks the 5-OH group) should be used as specificity controls (Table 8).

**TABLE 8:** PAINS/interference-risk checklist for genistein studies.

Assay type	Potential artefact	Required counter-screen	Recommended orthogonal validation
Fluorescence ROS (DCFH2-DA)	Direct probe reduction by genistein phenolics	Genistein-only + DCFH2-DA blank	HPLC-based ROS quantification; EPR
MTT/XTT/WST-1 viability	Direct tetrazolium reduction	Genistein-only blank at each concentration	Trypan blue exclusion; LDH release; ATP luminescence
Fluo-4/calcium imaging	Intrinsic fluorescence overlap; Ca2+ chelation	Genistein-only fluorescence control	Ratiometric dyes (Fura-2); calcium electrode
JC-1/mitochondrial potential	Fluorescence interference; aggregation quenching	Genistein-only + JC-1 control; CCCP positive	TMRM (non-ratiometric); Seahorse respirometry
Annexin V-FITC apoptosis	Fluorescence interference; membrane perturbation	Genistein-only + Annexin control	TUNEL (non-FITC); cleaved caspase-3 Western blot
Kinase inhibition assay	Colloidal aggregation (false-positive inhibition)	+/− 0.01% Triton X-100 detergent control	Kinase binding assay (SPR, ITC); CETSA
Enzyme inhibition (cell-free)	Metal chelation (metalloenzymes); aggregation	Add excess metal (Zn2+, Fe2+); detergent control	Genetic knockdown/knockout controls
Antioxidant (ORAC, DPPH, FRAP)	Direct radical scavenging (physicochemical property)	Compare to reference antioxidants; concentration response	ARE-luciferase reporter (cell-based)
Iron/ferroptosis assays	Fe2+ chelation by catechol-like motif	Add excess Fe2+; measure labile iron pool; Lipid peroxidation (C11-BODIPY)	GPX4 Western blot
Nitric oxide (Griess, DAF-FM)	Nitrite interference; fluorescence quench	Genistein + nitrite standard; NO donor control	eNOS/iNOS activity (citrulline assay)

EPR, electron paramagnetic resonance; SPR, surface plasmon resonance; ITC, isothermal titration calorimetry; CETSA, cellular thermal shift assay; ARE, antioxidant response element.

## 7 Drug interaction profile

Ischemia-reperfusion injury commonly occurs in perioperative, cardiovascular, stroke, renal-transplant, and critically ill settings where patients are frequently exposed to polypharmacy. Genistein has the potential to interact with multiple drug-metabolizing enzymes and transporters, a consideration that is central to translational interpretation but has not been addressed in the genistein IRI literature.

### 7.1 Pharmacokinetic interactions

CYP3A4/5: Genistein is a moderate inhibitor of CYP3A4 (IC50 approximately 10–25 μM in human liver microsomes) and may also induce CYP3A4 through PXR activation at lower concentrations. CYP3A4 metabolizes approximately 50% of clinically used drugs, including many sedatives (midazolam), calcium-channel blockers (nifedipine, verapamil), selected statins (atorvastatin, simvastatin; not pravastatin or rosuvastatin), immunosuppressants (tacrolimus, cyclosporine, sirolimus), and some direct oral anticoagulants (rivaroxaban, apixaban). The net effect (inhibition versus induction) likely depends on genistein concentration, duration of exposure, and CYP3A4/5 genetic polymorphisms.

CYP2C9: Genistein inhibits CYP2C9 (IC50 approximately 15–40 μM). CYP2C9 substrates include warfarin (S-enantiomer), many NSAIDs (celecoxib, diclofenac, ibuprofen), sulfonylureas, and phenytoin. Genistein-mediated CYP2C9 inhibition could potentiate anticoagulant effects and increase bleeding risk.

CYP2C8: Genistein may inhibit CYP2C8, which metabolizes paclitaxel, repaglinide, and some anti-inflammatory drugs. Clinical significance is likely moderate but not established. CYP1A2 is weakly inhibited by genistein; clinical significance is likely limited.

UGTs: Genistein itself is extensively glucuronidated by UGT1A1, UGT1A8, and UGT1A9. Competitive inhibition of these UGTs by genistein could alter the clearance of drugs that undergo glucuronidation, including acetaminophen, morphine, and some NSAIDs.

Transporters: Genistein is a substrate and inhibitor of BCRP/ABCG2 (IC50 approximately 25 μM) and P-glycoprotein (P-gp/ABCB1; IC50 approximately 50–100 μM). BCRP is expressed at the apical membrane of enterocytes, the bile canalicular membrane of hepatocytes, the proximal tubular epithelium in the kidney, and the luminal side of brain capillary endothelial cells (BBB). Inhibition of BCRP or P-gp could alter the absorption, tissue distribution, and elimination of drugs with narrow therapeutic indices, including digoxin, dabigatran etexilate (a P-gp substrate), immunosuppressants, and certain anticancer agents. OATP and OAT transporter interactions have been less characterized.

### 7.2 Pharmacodynamic interactions

Beyond metabolic interactions, genistein’s pharmacodynamic profile overlaps with multiple drug classes commonly used in IRI settings. Genistein has been reported to inhibit platelet aggregation in some studies (possibly through tyrosine kinase inhibition and cAMP elevation), and its antioxidant/anti-inflammatory effects may theoretically add to or confound the effects of aspirin, clopidogrel, heparin/warfarin, and direct oral anticoagulants. The vasodilatory properties of genistein (via eNOS/NO and calcium-channel modulation) may have additive effects with antihypertensive drugs (ACE inhibitors, ARBs, CCBs, beta-blockers). Statins have pleiotropic anti-inflammatory and antioxidant effects that overlap with genistein’s mechanisms, making it difficult to parse additive, synergistic, or redundant effects. NSAIDs and corticosteroids share anti-inflammatory endpoints with genistein; combined use may increase gastrointestinal, renal, and cardiovascular risks.

As a phytoestrogen, genistein may theoretically interfere with tamoxifen (competitive ER binding), aromatase inhibitors (exogenous estrogenic activity), and other endocrine therapies. This is particularly relevant for patients with hormone-sensitive cancers who may also be at risk for IRI. Soy isoflavones have been reported to affect thyroid function in iodine-deficient individuals and may interfere with levothyroxine absorption. Genistein’s modulation of GABA-A, glycine, and NMDA receptors and effects on vascular tone could interact with volatile and intravenous anesthetic agents. These considerations are summarized in Table 9.

**TABLE 9:** Genistein drug interaction risk profile.

Interaction domain	Affected target	Relevant drug classes	Evidence level	Potential clinical significance
CYP3A4 inhibition/induction	CYP3A4	Statins (atorvastatin, simvastatin), CCBs, immunosuppressants, selected DOACs	*In vitro*; *in vivo* unknown	Moderate
CYP2C9 inhibition	CYP2C9	Warfarin, NSAIDs, sulfonylureas	*In vitro* only	Moderate-High (warfarin)
CYP2C8 inhibition	CYP2C8	Paclitaxel, repaglinide	*In vitro* only	Low-Moderate
UGT competition	UGT1A1, 1A8, 1A9	Acetaminophen, morphine, some NSAIDs	*In vitro* only	Low-Moderate
BCRP/P-gp inhibition	BCRP/ABCG2, P-gp/ABCB1	Digoxin, dabigatran, immunosuppressants, anticancer drugs	*In vitro*	Moderate
Antiplatelet overlap	Platelet aggregation	Aspirin, clopidogrel, anticoagulants	Limited *in vivo* data	Unknown
Estrogen receptor competition	ER type	Relevant Drug Classes	Evidence Level	Potential Clinical Significance
Estrogen receptor competition	ERalpha/ERbeta	Tamoxifen, aromatase inhibitors, HRT	*In vitro* competitive binding	High (hormone-sensitive cancers)
Thyroid interference	T4 absorption, TPO	Levothyroxine	Soy reports; genistein-specific limited	Moderate (iodine-deficient patients)
Antihypertensive additivity	eNOS/NO, Ca2+ channels	ACE inhibitors, ARBs, CCBs, beta-blockers	No interaction studies	Unknown (likely low)
Anesthetic receptor modulation	GABA - A, NMDA, glycine	Volatile anesthetics, propofol	Limited	Unknown

CCBs, calcium-channel blockers; DOACs, direct oral anticoagulants; HRT, hormone replacement therapy; TPO, thyroid peroxidase; ACE, angiotensin-converting enzyme; ARBs, angiotensin receptor blockers.

## 8 Comparison with established IRI-protective strategies

To contextualize genistein’s translational potential, it is instructive to compare it with established and emerging IRI-protective strategies. Ischemic conditioning (preconditioning, postconditioning, remote conditioning) represents the most mechanistically validated and clinically tested strategy, with modest cardioprotective and neuroprotective signals in some clinical trials. The mechanistic overlap with genistein (both engage PI3K/Akt, mKATP, PKC, and mitochondrial pathways) suggests potential additive or synergistic effects that have not been explored.

Mitochondrial permeability transition pore inhibitors (cyclosporine A and non-immunosuppressive derivatives NIM811, Debio025) showed initial promise but failed in large phase III trials (CIRCUS, CYCLE), serving as a cautionary example for genistein: targeting a convergent IRI node does not guarantee clinical success. Nrf2 activators (bardoxolone methyl, sulforaphane, dimethyl fumarate) have progressed to clinical testing; bardoxolone showed renal benefit in phase II but was halted in phase III (BEACON) due to cardiovascular safety concerns. Genistein’s Nrf2 activation is weaker than these dedicated Nrf2 activators but possibly safer with chronic use.

Anti-inflammatory biologics (canakinumab, an anti-IL-1beta monoclonal antibody) reduced cardiovascular events in the CANTOS trial, supporting inflammation as a valid therapeutic target in IRI-relevant contexts. These agents are more potent and target-specific than genistein but are also more expensive and immunosuppressive. Antioxidants—including vitamin C, vitamin E, N-acetylcysteine, edaravone (approved for stroke in Japan), MitoQ, and MitoTEMPO—have mostly failed to translate despite strong preclinical data, providing a cautionary parallel for genistein’s antioxidant-based protection claims.

SGLT2 inhibitors (empagliflozin, dapagliflozin) have demonstrated remarkable cardiorenal protection in large outcome trials (EMPA-REG OUTCOME, DAPA-CKD, DAPA-HF), partly through IRI-relevant mechanisms including reduced oxidative stress, improved mitochondrial function, and anti-inflammatory effects. This drug class demonstrates that multi-target agents can succeed clinically, providing indirect validation for the multi-target strategy, though SGLT2 inhibitors have vastly stronger clinical evidence. GLP-1 receptor agonists (semaglutide, liraglutide) similarly reduce cardiovascular events and may protect against IRI through anti-inflammatory, antioxidant, and metabolic mechanisms. Adenosine pathway modulators (acadesine, ATL146e, regadenoson) showed preclinical promise but limited clinical success in IRI settings.

Organ-preservation solutions (University of Wisconsin solution, HTK, IGL-1, and machine perfusion strategies) represent the most clinically successful IRI interventions, particularly in transplantation. Genistein could be investigated as an additive to preservation solutions, a context in which high local concentrations can be achieved without systemic exposure constraints.

The clinical niche for genistein remains undefined. It is unlikely to compete with emergency interventions for acute myocardial infarction or stroke given the requirement for rapid onset and the uncertain pharmacokinetics of orally administered genistein. More plausible contexts include: (1) prophylactic supplementation before elective cardiac or vascular surgery; (2) additive to organ-preservation solutions for transplantation; (3) adjunctive therapy in settings where IRI is anticipated (e.g., planned percutaneous coronary intervention in high-risk patients); (4) long-term dietary supplementation for cardiovascular and cerebrovascular risk reduction in at-risk populations.

## 9 Translational barriers: Lessons from failed IRI interventions

The history of IRI drug development is dominated by translational failures. Hundreds of agents that showed robust protection in rodent IRI models subsequently failed in large clinical trials. Key lessons relevant to genistein include:

First, timing disconnect. Most preclinical IRI studies administer treatment before or immediately after ischemia, whereas clinical presentation delays (hours for stroke, hours for myocardial infarction) mean that treatment is initiated well after the therapeutic window has closed. Genistein studies are overwhelmingly pretreatment-based. Second, species differences. Rodent cardiomyocyte electrophysiology, coronary collateral circulation, immune system composition, drug metabolism, and infarct development kinetics differ substantially from those of large animals and humans. Genistein has been tested almost exclusively in rodents; porcine, canine, and non-human primate data are entirely absent. Third, concentration disconnect. As discussed in Section 3.1, *in vitro* concentrations (10–100 μM) far exceed *in vivo* free plasma concentrations (less than 0.1 μM with typical dietary intake; up to 1–5 μM with high-dose supplementation). Tissue-level genistein concentrations in IRI models have never been measured.

Fourth, comorbidity exclusion. Preclinical IRI studies almost always use young, healthy, genetically homogeneous animals. Human IRI patients are typically older with multiple comorbidities (diabetes, hypertension, atherosclerosis, chronic kidney disease) and concomitant medications that alter IRI pathophysiology and drug responses. Only one genistein study has been conducted in a diabetic IRI model (Rajput et al., 2017), and none in hypertensive, aged, or atherosclerotic models. Fifth, endpoint mismatch. Preclinical studies focus on acute histological and biochemical endpoints (infarct size, enzyme levels, inflammatory markers), whereas clinical trials use hard outcomes (mortality, major adverse cardiac events, stroke recurrence, graft survival, need for dialysis) assessed over months to years. Short-term biomarker improvements do not reliably predict long-term clinical benefit.

Sixth, sex as a biological variable. Most preclinical IRI studies use male animals only. Given genistein’s phytoestrogenic activity, sex-specific efficacy and safety cannot be assumed. Seventh, publication bias. Negative and neutral genistein IRI studies are rarely published, creating a literature skewed toward positive findings. Eighth, natural-product-specific limitations. Inconsistent compound purity, batch-to-batch variability in sourcing, inadequate blinding and randomization in animal studies, and absence of pre-registration of experimental protocols are prevalent concerns in the genistein IRI literature and contribute to reproducibility concerns. Ninth, industry independence. Most genistein IRI studies are academic; the absence of industry-standard pharmacokinetic profiling, toxicology, and formulation development limits translational readiness.

Acknowledging these barriers is essential for the credibility of the review and for setting realistic translational expectations. Without such a discussion, the translational claims regarding genistein as a clinically deployable cytoprotective agent remain premature.

## 10 Comorbidities in experimental IRI models

IRI severity and response to therapy are profoundly modified by comorbid conditions, yet the genistein IRI literature almost entirely neglects this dimension. Diabetes: hyperglycemia exacerbates oxidative stress, impairs ischemic preconditioning, alters KATP channel function, and reduces responsiveness to some cardioprotective agents. Only one genistein study has been conducted in a diabetic IRI model (global cerebral ischemia in streptozotocin-induced diabetic mice) (Rajput et al., 2017), and none in diabetic myocardial, renal, or hepatic IRI models. Hypertension: chronic hypertension induces vascular remodeling, endothelial dysfunction, and altered autoregulation. No genistein IRI studies have been conducted in hypertensive models (SHR, L-NAME, DOCA-salt, Goldblatt). Aging: aged animals show impaired stress responses, altered mitochondrial function, chronic low-grade inflammation (“inflammaging”), and reduced efficacy of ischemic conditioning. Genistein studies in aged rodents are limited to one cerebral IRI study in reproductively senescent female mice (Wang et al., 2020).

Obesity and metabolic syndrome: diet-induced obese models have not been used in genistein IRI research. Atherosclerosis: ApoE^−/−^ or LDLr^−/−^ atherosclerotic mouse models of IRI with genistein intervention have not been reported, despite genistein’s known anti-atherosclerotic effects documented with derivatives such as 7-difluoromethyl-5,4′-dimethoxygenistein (Zhao et al., 2009). Chronic kidney disease: the uremic milieu alters drug pharmacokinetics and IRI susceptibility; not studied with genistein. Concomitant medications: as discussed in Section 7, polypharmacy is the norm in IRI-prone patient populations, yet drug-drug interaction studies with genistein are entirely absent from the IRI literature. The absence of comorbidity-rich models is a major limitation of the genistein IRI evidence base. Future studies should prioritize efficacy testing in models that recapitulate the clinical demographics of IRI patients.

## 11 Separating genistein from related isoflavones and derivatives

The IRI literature on isoflavones includes studies on several structurally related compounds that should not be conflated with genistein without explicit qualification.

Genistein (4′,5,7-trihydroxyisoflavone) is the parent compound and the primary subject of this review. Genistein-3′-sodium sulfonate (GSS) is a water-soluble sulfonated derivative designed for parenteral administration, studied most extensively in cerebral IRI models (Xie et al., 2021; Xie et al., 2023; Li L. et al., 2017). Important differences from genistein include enhanced water solubility enabling intravenous administration, altered receptor-binding profile (the 3′-position modification may affect ER binding and kinase inhibition), potentially different biodistribution and BBB penetration, and a distinct metabolite profile. GSS-specific evidence should be clearly labeled as such and not presented as direct genistein evidence.

7- Difluoromethyl-5,4′-dimethoxygenistein (DFMG) is a synthetic genistein derivative with anti-atherosclerotic activity in rabbits (Zhao et al., 2009). Its pharmacological profile differs substantially from genistein. Biochanin A (5,7-dihydroxy-4′-methoxyisoflavone) is the 4′-O-methylated precursor of genistein, found in red clover and chickpeas. Biochanin A is metabolized to genistein *in vivo* by CYP-mediated demethylation, but it also has independent pharmacological activities (e.g., PPAR activation, different kinase inhibition profile). Biochanin A has been studied in cerebral, myocardial, and hepatic IRI models as a distinct compound and should not be presented as genistein evidence unless the interconversion is explicitly discussed. Daidzein (4′,7-dihydroxyisoflavone) lacks the 5-OH group; it shares some but not all of genistein’s activities and is generally less potent as a TKI and antioxidant. Soy extracts and mixed isoflavone preparations contain variable proportions of genistein, daidzein, glycitein, and their glucoside conjugates. Attributing effects of complex mixtures to genistein alone is methodologically inappropriate (Table 10).

**TABLE 10:** Genistein vs. related compounds in IRI research.

Compound	Key structural difference	IRI organs studied	Evidence distinct from genistein?	Should be discussed separately?
Genistein	Parent (5,7,4′-triOH)	Heart, brain, kidney, liver, intestine	N/A	Primary focus
Genistein-3′-sulfonate (GSS)	3′-sulfonate group	Brain, neonatal brain	Yes (solubility, PK, receptor profile)	Yes
DFMG (7-difluoromethyl)	7-CHF2, 5,4′-diOCH3	Atherosclerosis (not IRI *per se*)	Yes	Yes
Biochanin A	4′-OCH3 instead of 4′-OH	Brain, heart, liver	Yes (prodrug + independent activities)	Yes
Daidzein	Lacks 5-OH	Brain	Yes	Yes
Soy isoflavone extracts	Variable mixture	Heart, brain	Yes (cannot attribute to genistein)	Yes

## 12 Endocrine and safety considerations

As a phytoestrogen with binding affinity for both ERalpha and ERbeta (Ki approximately 0.1–1 μM, approximately 10- to 100-fold weaker than estradiol), genistein raises important endocrine safety considerations that have been insufficiently discussed in the IRI context.

Reproductive system: Chronic high-dose genistein in rodents has been associated with uterine hyperplasia, altered estrous cyclicity, and developmental effects when administered during critical windows (neonatal, peripubertal). Human data from soy-consuming populations and clinical trials of isolated genistein (54 mg/day for up to 3 years) have generally not shown increased risk of endometrial hyperplasia or cancer, though long-term safety data (greater than 5 years) are limited. Breast cancer: genistein’s effect on breast tissue is likely biphasic—at low (nanomolar) concentrations, it can stimulate ER-positive breast cancer cell proliferation *in vitro*; at high (micromolar) concentrations, it inhibits proliferation through TKI, epigenetic, and pro-apoptotic mechanisms. Epidemiological studies in Asian populations consuming lifelong soy suggest a protective association, but the safety of high-dose genistein supplementation in breast cancer survivors or high-risk women is not established.

Thyroid function: Soy isoflavones can inhibit thyroid peroxidase (TPO) *in vitro* and may exacerbate iodine deficiency-induced goiter. In iodine-sufficient individuals, moderate soy/genistein intake does not appear to significantly impair thyroid function, but monitoring is advisable for patients on thyroid replacement therapy or with pre-existing thyroid disease. Developmental exposure: neonatal genistein exposure in rodents has produced persistent alterations in reproductive development, immune function, and neurodevelopment. The relevance to short-term genistein administration for IRI in adult or elderly populations is unclear but warrants acknowledgment.

Male reproductive function: genistein has been associated with altered spermatogenesis and fertility in some animal studies at high doses, though human data are reassuring at dietary intake levels. Hormone-sensitive conditions: IRI can occur in patients with hormone-sensitive cancers (prostate, breast, endometrial), and in these populations, the risk-benefit ratio of phytoestrogen therapy must be carefully considered. IRI also occurs in perioperative and transplantation settings where estrogenic signaling may be undesirable (e.g., in patients with hormone-receptor-positive malignancies). The endocrine safety of genistein is likely dose-, duration-, age-, sex-, and comorbidity-dependent. Short-term (days to weeks) high-dose genistein in the perioperative or acute IRI setting may have a different safety profile than chronic dietary supplementation, but this has not been studied.

## 13 Dose translatability and treatment windows

A critical issue in the genistein IRI literature is the near-universal use of pretreatment paradigms. In the vast majority of reviewed studies, genistein was administered before the ischemic insult (anywhere from 30 min to 14 days prior). This is appropriate for mechanistic studies and for elective surgical contexts, but has limited relevance to unplanned clinical IRI events such as acute myocardial infarction, ischemic stroke, trauma, or cardiac arrest.

Pretreatment and post-treatment paradigms must be clearly separated. Peri-reperfusion administration (genistein given just before or at the onset of reperfusion) has shown infarct size reduction in cardiac IRI models (Ji et al., 2004), suggesting a peri-reperfusion window. In cerebral IRI, GSS administered immediately after reperfusion (within 30 min to 2 h) reduced infarct volume and improved outcomes (Xie et al., 2021; Xie et al., 2023; Li L. et al., 2017), providing the strongest evidence for post-treatment efficacy, though the time window is narrow. In renal IRI, all studies have used pretreatment; no post-ischemia genistein administration data are available.

Regarding dose translation, a formal allometric dose-translation analysis is lacking in the genistein IRI literature. Based on the FDA body surface area (BSA) normalization method, typical rodent genistein doses of 10–50 mg/kg/day (i.p. or p.o.) would correspond to human equivalent doses (HED) of approximately 0.8–4 mg/kg/day, or 50–280 mg/day for a 70-kg adult. These doses are achievable with oral supplementation (commercial genistein supplements provide 50–300 mg per dose), but free aglycone plasma concentrations at these doses remain in the low nanomolar range, far below the micromolar concentrations used in virtually all *in vitro* studies. High-dose intraperitoneal or pretreatment-only models should not be presented as directly clinically actionable without explicit qualification (Table 11).

**TABLE 11:** Dose translatability for key genistein IRI studies.

Organ/Model	Effective rodent dose	Route	Timing	HED (mg/day, 70 kg)	Clinically plausible?
Cardiac (rabbit)	1 mg/kg i.v	i.v	Peri-reperfusion	∼56 mg i.v	Requires parenteral formulation; unlikely for acute MI
Cerebral (rat)	10 mg/kg i.p. × 14 days	i.p	Pretreatment (14 days)	∼112 mg/day p.o	Feasible for prophylaxis; not for acute stroke
Renal (rat)	50 mg/kg p.o. × 3 days	p.o	Pretreatment (3 days)	∼560 mg/day p.o	High-dose supplement; feasible for pre-transplant
Cerebral GSS (rat)	15–30 mg/kg i.v	i.v	Post-reperfusion	170–340 mg i.v	Requires parenteral GSS formulation development

## 14 Conclusion and translational roadmap

### 14.1 Summary of evidence

Genistein, a naturally derived isoflavone compound, has demonstrated protective effects in preclinical IRI models across multiple organs (heart, brain, kidney, liver, intestine, testis). The mechanistic evidence encompasses antioxidant (Nrf2/HO-1, ADORA2A-cAMP, direct radical scavenging), anti-inflammatory (NF-kappaB, JAK2/STAT3, alpha7nAChR, NLRP3 inflammasome), anti-apoptotic (PI3K/Akt, SIRT1/p53 deacetylation, Bcl-2 family modulation, ERK1/2 versus JNK/p38 MAPK regulation), and mitochondrial-protective (mPTP inhibition, membrane potential stabilization, mKATP channel modulation) pathways. Among these, the strongest evidence (Level A: causal validation by genetic or pharmacological tools) supports the involvement of Nrf2/HO-1 in cerebral IRI (Nrf2 siRNA), SIRT1/p53 (EX527/siRNA) and ADORA2A-cAMP-PK (SCH58261) in renal IRI, PI3K/Akt (LY294002) in cardiac and renal IRI, and alpha7nAChR-JAK2/STAT3 (MLA) in cerebral IRI with GSS. Evidence for hepatic, intestinal, and other organ protection remains largely correlative (Level C).

### 14.2 Critical gaps and limitations

Despite the breadth of preclinical data, significant translational gaps preclude clinical readiness: (1) pharmacokinetic disconnect between *in vitro* effective concentrations (10–100 μM) and *in vivo* free plasma aglycone levels (less than 0.1 μM); (2) unaddressed PAINS and assay-interference liability in all reviewed studies; (3) absence of large-animal (porcine, canine, non-human primate) data; (4) near-complete exclusion of comorbidities (diabetes, hypertension, aging, atherosclerosis, obesity); (5) near-universal reliance on pretreatment paradigms with limited post-reperfusion efficacy data; (6) complete absence of formal drug-drug or drug-disease interaction studies in IRI-relevant contexts; (7) unresolved endocrine safety in hormone-sensitive populations; (8) lack of an optimized clinical formulation for IRI indications; (9) zero human IRI clinical trials; and (10) publication bias and natural-product-specific limitations (inconsistent purity, batch variability, inadequate blinding/randomization).

### 14.3 Translational roadmap

We propose the following prioritized steps to advance genistein from preclinical promise toward clinical investigation. Phase 1 (Strengthen the Preclinical Foundation): standardize IRI models across laboratories; perform causal validation for each major mechanistic claim using genetic knockout, siRNA, CRISPR interference, or selective pharmacological inhibitors with rescue experiments; include orthogonal, non-fluorescence-based assays for ROS, viability, and mitochondrial function to address PAINS concerns; measure free genistein concentrations in plasma and target tissues under efficacy dosing regimens; test post-reperfusion administration paradigms to define therapeutic windows; and include both sexes and at least one comorbidity model (e.g., diabetic, aged) in efficacy studies.

Phase 2 (Address Formulation and Safety): develop and characterize an injectable genistein formulation (nanoparticle, liposomal, or cyclodextrin-based) with defined pharmacokinetics, stability, sterility, and endotoxin specifications; conduct formal ADME and toxicology studies in a large-animal model; perform *in vitro* drug-interaction screens against major CYPs, UGTs, and transporters in human hepatocytes and recombinant enzyme systems; and conduct reproductive and endocrine safety studies appropriate to the target clinical population.

Phase 3 (Early Clinical Contexts): The most plausible initial clinical contexts for genistein are those in which IRI is anticipated and prophylactic or peri-procedural administration is feasible. Renal transplantation—adding genistein to organ-preservation solutions and administering to recipients perioperatively—represents the strongest case, given the Level A mechanistic evidence for genistein in renal IRI and the well-defined clinical endpoint of delayed graft function (DGF). Elective cardiac surgery with cardiopulmonary bypass, elective carotid endarterectomy or intracranial aneurysm surgery with temporary vessel occlusion, and planned percutaneous coronary intervention in high-risk patients are additional plausible contexts. Only after safety and feasibility are demonstrated in these controlled settings should investigation expand to acute, unplanned IRI events such as emergency PCI for STEMI or acute ischemic stroke.

In conclusion, genistein is a pharmacologically rich natural product whose multi-target profile aligns rationally with the multi-factorial pathophysiology of IRI. However, the current evidence base, while broad, is insufficiently rigorous to support claims of clinical readiness. We caution against presenting genistein as a mechanistic panacea in which every canonical cytoprotective pathway is activated or inhibited in the expected direction, and instead encourage a nuanced, evidence-tiered approach that distinguishes robustly validated mechanisms from associative biomarker changes. With coordinated preclinical and early clinical efforts addressing the identified gaps, genistein may find a defined niche in the prevention and treatment of IRI, particularly in elective surgical and transplantation settings where the therapeutic window can be anticipated.
